# Predicting the conformational flexibility of antibody and T cell receptor complementarity-determining regions

**DOI:** 10.1038/s42256-025-01131-6

**Published:** 2025-10-16

**Authors:** Fabian C. Spoendlin, Monica L. Fernández-Quintero, Sai S. R. Raghavan, Hannah L. Turner, Anant Gharpure, Johannes R. Loeffler, Wing K. Wong, Alexander Bujotzek, Guy Georges, Andrew B. Ward, Charlotte M. Deane

**Affiliations:** 1 Department of Statistics, University of Oxford, Oxford, UK; 2 Department of Integrative Structural and Computational Biology, The Scripps Research Institute, San Diego, CA USA; 3 Large Molecule Research, Roche Pharma and Early Development, Roche Innovation Center Munich, Penzberg, Germany

**Keywords:** Biologics, Structural biology

## Abstract

Many proteins are highly flexible and their ability to adapt their shape can be fundamental to their functional properties. For example, the flexibility of antibody complementarity-determining region (CDR) loops influences binding affinity and specificity, making it a key factor in understanding and designing antigen interactions. With methods such as AlphaFold, it is possible to computationally predict a single, static protein structure with high accuracy. However, the reliable prediction of structural flexibility has not yet been achieved. A major factor limiting such predictions is the scarcity of suitable training data. Here we focus on predicting the structural flexibility of functionally important antibody and T cell receptor CDR3 loops. To this end, we constructed ALL-conformations by extracting CDR3s and CDR3-like loop motifs from all structures deposited in the Protein Data Bank. This dataset comprises 1.2 million loop structures representing more than 100,000 unique sequences and captures all experimentally observed conformations of these motifs. Using this dataset, we develop ITsFlexible, a deep learning tool with graph neural network architecture. We trained the model to binary classify CDR loops as ‘rigid’ or ‘flexible’ from inputs of antibody structures. ITsFlexible outperforms all alternative approaches on our crystal structure datasets and successfully generalizes to molecular dynamics simulations. We also used ITsFlexible to predict the flexibility of three CDRH3 loops with no solved structures and experimentally determined their conformations using cryogenic electron microscopy.

## Abstract (web summary)

ALL-conformations, a dataset capturing the full range of experimentally observed conformations of CDR loops, T cell and antibody regions interacting with antigen targets, is introduced. ITsFlexible—a deep learning tool trained on this new dataset—advances predictions of immune receptor structural dynamics.

## Main

Many proteins are flexible molecules that adopt several stable structures, termed conformations, and transitions between them can be fundamental for their function^1,2^. Antibodies and T cell receptors (TCRs) primarily engage their targets through six loop motifs called the complementarity-determining regions (CDRs)^3^. Structural flexibility of CDRs has been linked to several key functional properties. For some antibodies, conformational changes are known to be required for antigen recognition^4^. The ability of an antigen receptor to adopt multiple conformations has also been associated with polyspecificity as different structural states allow for greater variability in the recognized antigens^5,6^. Furthermore, flexibility has an effect on binding affinity as it directly impacts the entropic costs of antigen binding^7^ and rigidification has been observed as one of the natural mechanisms used to increase affinity^8,9^.

Specificity and affinity are two critical properties of antibody and TCR therapeutics. To maximize target interactions and minimize off-target interactions, a therapeutic should have high affinity and specificity^10^. This suggests that therapeutics should have a preference towards rigidity. However, there is some evidence indicating that conformational flexibility allows a better recognition of mutated antigen variants and could be desired when designing broadly neutralizing antibodies^11,12^. In either case, a method predicting CDR flexibility would allow both an enhanced investigation of antibody function and the potential to tune the desired therapeutic properties.

Computational predictions of a single, static structure of a protein from its sequence is now considered a routine task (for example, refs. ^13–15^), and more recently developed tools are also showing promise at protein complex prediction (for example, refs. ^16,17^). However, predicting structures of more than one conformational state remains challenging. One factor that has limited progress in conformation prediction is the scarcity of suitable data.

Evidence on conformational flexibility can currently be obtained from several experimental sources. Nuclear magnetic resonance spectroscopy and hydrogen–deuterium exchange mass spectrometry can be used to measure the protein dynamics in solution, although they do not typically provide atomically resolved flexibility information (see ref. ^18^ for a review of nuclear magnetic resonance and ref. ^19^ for hydrogen–deuterium exchange methods). X-ray crystallography is the standard method used to obtain the high-resolution structures of conformational states. These can be captured by solving separate structures of the same protein under different conditions. Because multiple structures must be available, the number of proteins for which flexibility can be assessed from the crystallographic data is much smaller than the total number of solved structures. Crystal structures have been used to explore the flexibility of specific loop types^20^ or for case studies of full-length proteins^21–23^, but there has been little systematic mining of the Protein Data Bank (PDB) for all instances of the same sequence representing alternative conformations^24^.

Molecular dynamics (MD) simulations provide a computational way to generate conformational ensembles. MD simulations are computationally expensive, and therefore, even the largest databases of standardized MD simulations are not yet sufficient for training machine learning models^25^.

Despite these data challenges, a number of methods have been developed that attempt to predict structures of protein conformational ensembles. Recent approaches have concentrated on modifying the AlphaFold2 (AF2) inference procedure to increase the diversity of outputs (for example, refs. ^22,23,26–30^). These methods target multiple sequence alignment (MSA), one of the main inputs of AF2, from which co-evolutionary signals are extracted to infer protein residues probably in close proximity. In theory, the MSA should contain co-evolutionary information for all conformational states. Deconvolving these signals is generally attempted by reducing the depth of the MSA, for example, through random subsampling^22^ or sequence clustering^26^. More recently, a range of methods were specifically trained for the task of conformation prediction (for example, refs. ^31–37^). These generally take the form of generative protein structure prediction models trained on the PDB and a small number of MD simulations.

Detailed evaluation of conformation prediction tools is complicated by data scarcity, but available evidence suggests that reliable predictions are not yet possible. Many methods have only been evaluated on one or a few case studies, which may not accurately reflect their predictions across diverse sets of proteins^21,28–30,33^. The evaluation of some methods on slightly larger test sets show that the diversity of predicted structures is generally increased; however, the conformational landscape is not covered with high accuracy^36^. The prediction of the conformational states of antibody and TCR CDRs specifically has only been assessed on a handful of case studies^21^.

**Fig. 1:**
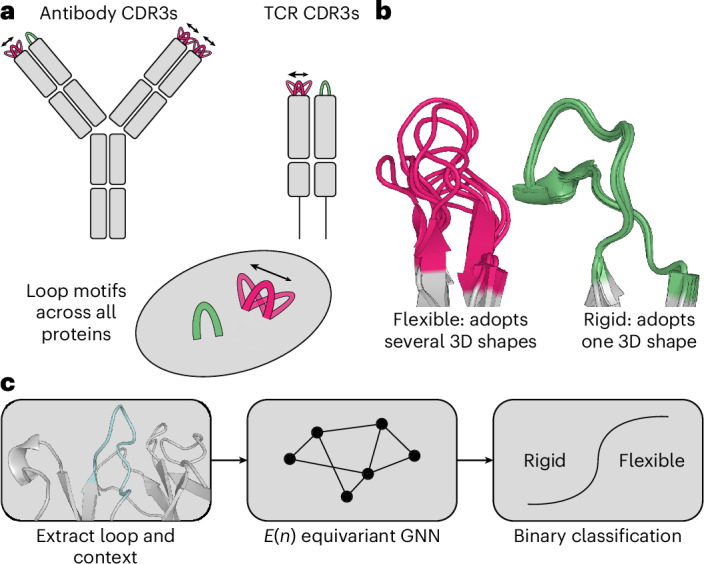
Overview of ALL-conformations and ITsFlexible. **a**, ALL-conformations is a dataset that contains the crystal structures of antibody CDR3s, TCR CDR3s and CDR-like loop motifs across all proteins. The dataset captures all the observed conformational states of such loops. **b**, Loops are labelled as either flexible (if they are observed in more than one conformation) or rigid (if evidence suggests that they adopt a single conformation). We define a conformation by structural similarity and use an RMSD of 1.25 Å as a threshold to separate states. **c**, Flowchart detailing the ITsFlexible method predicting the conformational flexibility of CDR loops. The structure and sequence of a loop (cyan) and its context (grey) are extracted from a PDB file, and a graph representation is generated. A graph neural network (GNN) classifies loops as conformationally flexible or rigid.

In this work, we focus on the flexibility of the functionally important antibody and TCR CDR3s. In an attempt to address the issue of data scarcity, we consider loop motifs with the same secondary structure pattern, defined as loops bounded by two antiparallel β-strands, across all proteins. Through a systematic mining of the PDB^38^ and antibody- and TCR-specific databases^39,40^, we created antibody-like loop conformations (ALL-conformations)—a dataset containing 1.2 million crystal structures of loops with 100,000 unique sequences. The dataset captures all the experimentally observed conformations of loop motifs found between pairs of antiparallel β-strands including both antibody and TCR CDR3s (Fig. 1a). We analysed the structural flexibility in the ALL-conformations set and label more than 20,000 unique loop sequences by their ability to undergo conformational changes (Fig. 1b). Using these data, we built an Immunoglobulins and TCRsFlexibility classifier (ITsFlexible)—a method that classifies whether CDR3s are rigid (adopt a single conformation) or exhibit flexibility (transition between multiple states) (Fig. 1c). ITsFlexible predicts the flexibility of CDR3s evaluated in ensembles of crystal structures with high accuracy and achieves state-of-the-art performance. The model also effectively generalizes to a test set derived from MD simulations. Furthermore, we used ITsFlexible to predict the flexibility of three CDRH3 loops with no solved structures and used cryogenic electron microscopy (cryo-EM) to experimentally determine their conformations. These experiments showed that two of the three model predictions were correct.

ALL-conformations is released on Zenodo (10.5281/zenodo.15784241)^41^, and ITsFlexible is available on GitHub (https://github.com/oxpig/ITsFlexible) and Zenodo (10.5281/zenodo.16891380)^42^.

### Results

#### ALL-conformations dataset

ALL-conformations is a dataset that captures the conformational flexibility of loop motifs bounded by two antiparallel β-strands, which included five subsets, antibody CDRH3s and CDRL3s, TCR CDRB3s and CDRA3s and loop motifs across all proteins in the PDB (Supplementary Fig. 4). The dataset collects available crystal structures of such loops and contains the structures of all experimentally observed conformational states.

Extracting CDR3 structures from structural antibody database (SAbDab)^39^ and structural T cell receptor database^40^ and loop motifs from the PDB^38^, we obtain a total of more than 1.2 million examples with more than 100,000 unique sequences (Table 1). The PDB set contains loops between 1 and 87 residues; however, it is heavily enriched for shorter loops (Supplementary Fig. 5). Length distributions are different for CDR3s with a peak of around 10 to 15 residues and no loops shorter than 4 amino acids (Supplementary Fig. 6). We found that a large number of loop sequences are represented by multiple crystal structures (Supplementary Figs. 5 and 6).

**Table 1:** Number of structures and sequences in ALL-conformations

Dataset	Structures	Unique loop/loop + anchors/Fv sequences	Loop/loop + anchors/Fv sequences with multiple structures
PDB set	1,208,000/-	99,000/162,000/-	69,000/112,000/-
Antibodies			
CDRH3	6,401	2,190/2,190/2,477	1,372/1,372/1,460
CDRL3	6,401	1,762/1,776/2,366	1,147/1,150/1,432
TCRs			
CDRB3	691	197/197/234	137/137/144
CDRA3	691	192/196/233	134/137/151

The loops were classified as rigid, flexible or unknown (Methods). Loops for which multiple conformations are observed in the crystal structures were labelled as flexible. Here we define a conformation as a cluster in which the pairwise root mean square deviation (RMSD) of any member is below a threshold of 1.25 Å. This approach was chosen because it was previously shown to provide a good functional clustering of antibodies^43^. A conformation should, therefore, reflect a functionally distinct structural state. It is not possible to guarantee that all loops only observed in a single conformation are indeed rigid as there always remains a possibility that additional conformational states have not yet been captured. To address this issue, we only label loops as rigid if they adopt the same conformation in more than five structures. This should ensure that the set is heavily enriched for loops with a single accessible conformation. Using these definitions, we identified more than 16,000 rigid and 4,000 flexible loops (Supplementary Table 5). For the evaluation of the impact of RMSD threshold on the proportion of two classes, see Supplementary Fig. 10 and Supplementary Section 2.3.

#### Predicting the flexibility of CDR-like protein loop motifs

The ALL-conformations set was used to train ITsFlexible, a model that predicts protein loop flexibility. ITsFlexible is a graph neural network (Supplementary Fig. 1) that binary classifies loops if they can occupy multiple conformations (flexible) or adopt a single stable state (rigid) from inputs encoding the sequence and structure of a loop and its structural context (Methods).

**Fig. 2:**
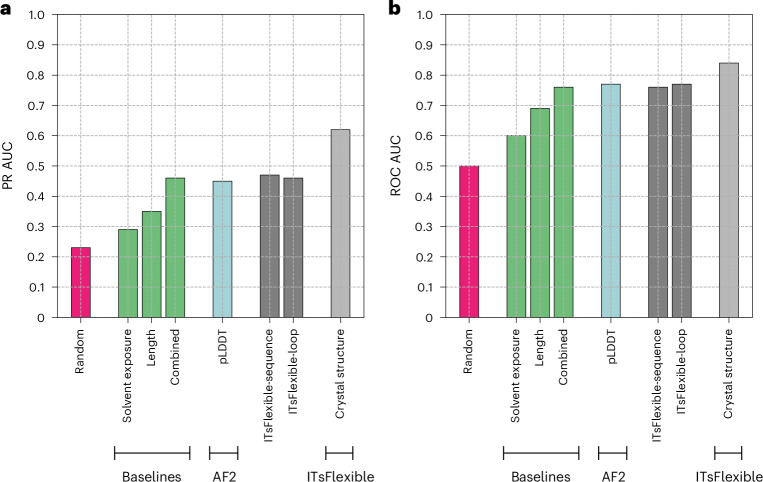
ITsFlexible performance evaluated on the PDB test set. **a**,**b**, Classification on the PDB test set containing 2,845 loop motifs is evaluated with metrics of PR AUC (**a**) and ROC AUC (**b**). ITsFlexible performance (light grey) is compared with random classification (red), three biophysical baselines (green), an AF2 pLDDT-based model (blue) and two ITsFlexible versions with input ablations (dark grey). Exact PR AUC and ROC AUC values are presented in Supplementary Tables 9 and 10.

We trained and evaluated ITsFlexible on the PDB set of ALL-conformations, containing loop motifs observed across any protein. The data split was performed based on sequence identity and test set loops had a maximum of 80% sequence identity with length-matched loops in the training and validation sets. Classifier performance was compared with random classification, three baseline models and a zero-shot flexibility prediction workflow based on AF2 predicted local distance difference test (pLDDT)^13^ (Fig. 2). Random classification shows validation metrics when randomly classifying a dataset with the given proportion of labels. The baselines predict flexibility from biophysical features such as loop length and solvent exposure, which have previously been shown to influence loop dynamics^20,44^. Long loops contain more bonds around which they can rotate and solvent exposure reduces steric hindrance, which restricts conformational rearrangements. pLDDT is a residue-level confidence score and provides a good indicator of disordered protein regions^13^. As the test set contains several structures containing each loop, an ITsFlexible prediction was made for all of them and the metrics were calculated based on the mean prediction score.

ITsFlexible is able to classify loop flexibility (area under the precision–recall curve (PR AUC) of 0.62 and receiver operating characteristic area under the curve (ROC AUC) of 0.84) and outperforms our baselines and zero-shot classifier. We investigated features predictive of loop flexibility through the ablation of model inputs. ITsFlexible-loop is similar to our default model but inputs are reduced to the structure and sequence of only the loop itself (Supplementary Section 1.1). ITsFlexible-sequence is a convolutional-neural-network-based model trained on a sequence encoding loops (Supplementary Section 1.1). ITsFlexible-loop and ITsFlexible-sequence achieve similar performance. Both outperform the relevant baseline (only baseline length is relevant as solvent exposure depends on the structural context, which both models do not consider) but are less predictive than ITsFlexible (Fig. 2).

These results give some indication of the factors that determine the conformational flexibility of protein loops. It is well known that longer and solvent-exposed loops tend to be more flexible than shorter and buried ones^20,44^. Although our findings agree with these general trends, we also show that the loop sequence is more predictive of flexibility than length alone, suggesting that the sequence of a loop impacts its ability to adopt multiple conformations. A large boost in performance was achieved by encoding the structural context of the loop motif. In line with previous evidence from MD studies^44^, this highlights that the interactions of a loop with its context within the protein are important determinants of its conformational dynamics.

#### ITsFlexible is highly predictive of CDR3 flexibility

We next investigated the ability of ITsFlexible, trained on general proteins loops, to predict the flexibility of antibody and TCR CDR3s. The model was evaluated on the CDR3 sets of ALL-conformations, which were designed to have no overlap, defined as more than 80% aligned sequence identity, with the training and validation sets.

We initially evaluated ITsFlexible using crystal structures as the input. In addition to the baselines and the pLDDT-based predictor introduced in the previous section, we evaluated two additional zero-shot flexibility predictors. The first workflow predicts flexibility based on the diversity in CDRs modelled by multiple AF2 runs with subsampled MSAs (Methods). The second workflows uses the residue-level confidence score (root mean square predicted error (RMSPE)) of ABodyBuilder2 (ABB2), an antibody-specific structure predictor^45^.

**Fig. 3:**
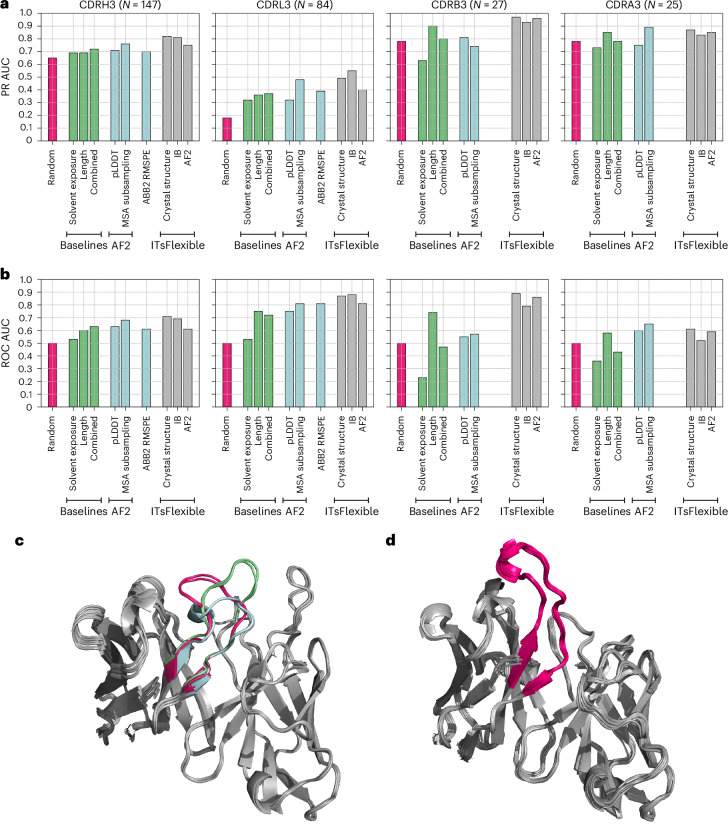
ITsFlexible performance evaluated on the CDR test sets. **a**,**b**, Classification on the four test sets is evaluated with metrics of PR AUC (**a**) and ROC AUC (**b**). ITsFlexible performance from inputs of crystal structures, IB and AF2 models (light grey) is compared with random classification (red), three biophysical baselines (green) and three zero-shot models based on the outputs of protein structure prediction tools (blue). Exact PR AUC and ROC AUC values are presented in Supplementary Tables 9 and 10. **c**,**d**, Example of antibodies predicted to be flexible and rigid by ITsFlexible. **c**, Overlay of six structures of the same antibody Fv with CDRH3 predicted to be flexible. Crystal structures indicated that the CDRH3 (highlighted in colour) adopts three different conformations (red, blue and green). **d**, Overlay of 22 structures of the same antibody Fv with CDRH3 predicted to be rigid. The CDRH3 (red) occupies the same conformations in all 22 structures.

ITsFlexible proved to be highly predictive of antibody and TCR CDR3 flexibility (Fig. 3). Our method outperforms the biophysical baselines and zero-shot classifiers in nearly all CDR test sets. The only exception is the CDRA3 set in which the MSA subsampling approach surpasses ITsFlexible by a narrow margin. However, ITsFlexible substantially outperforms all other methods on CDRH3s, the largest and, therefore, the most representative set, and is the only method that consistently achieves high predictive accuracy across all four test sets. We also point out that ITsFlexible was evaluated with the most stringent train–test split compared with the zero-shot models. Specifically, CDRs in the ITsFlexible training set were filtered by 80% sequence identity to the test sets, whereas 100% sequence identity was used for ABB2 and the AF2 training set may even contain test set overlaps.

We conducted a more detailed investigation of the ABB2 confidence score. This analysis revealed that RMSPE is potentially more indicative of the number of times a certain antibody occurs in the training data rather than flexibility (Supplementary Section 2.4).

Comparing the classification performance between the four CDR sets (using ROC AUC), we observed the best results for L3s and B3s with slightly lower values for H3s and A3s. Antibody H3s have higher sequence diversity than L3s, which is expected to make classification more difficult. The lower performance for A3s could be linked to the findings of a different pattern of genetic diversity compared with B3s^46^. However, we also highlight the small size of the A3 set, which may result in a less accurate estimation of performance.

Furthermore, we analysed the consistency of ITsFlexible predictions when using different structures containing the same loop as the model input. The results show that variations in prediction scores are generally small, demonstrating consistency (Supplementary Fig. 7).

As structural data are only available for a subset of known antigen receptor sequences^47,48^, ITsFlexible was additionally tested using predicted structural models from ImmueBuilder (IB) and AF2 as inputs. On the antibody CDR sets, similar performance is observed for predictions from crystal structures and IB models and slightly worse predictions are made from AF2 models. Although there is no clear correlation between model quality and the error in the ITsFlexible prediction, we observed that higher prediction errors tend to occur at lower model quality (Supplementary Fig. 8). The difference in classification performance may, therefore, be tied to the lower quality of AF2 compared with IB models of our test set (Supplementary Table 7). On the TCR sets, predictions from AF2 models are marginally better in PR AUC than from IB models with a larger difference in ROC AUC. As for the antibody sets, we observed a lower model accuracy and a larger error in ITsFlexible prediction score for AF2 (Supplementary Table 7). However, for TCRs, this does not deteriorate the classification metrics.

Throughout the analysis in this paper, we made the following assumptions to determine CDR flexibility. CDR3s are defined as International ImMunoGeneTics (IMGT) residues 107–116 (Methods and Supplementary Fig. 4), structural diversity is calculated as the loop RMSD after alignment on the loop residues and a conformation is defined as a structural cluster with pairwise RMSD between any member below 1.25 Å. Additional analysis was performed on datasets with CDR3s defined by their exact secondary structure (Supplementary Table 8), flexibility calculated by alignment on the framework residues (Supplementary Fig. 9) and different choices of RMSD thresholds (Supplementary Fig. 10). We found that ITsFlexible’s high predictive accuracy remains qualitatively similar for the first two experiments, whereas the choice of threshold impacts classification to a minor extent.

#### ITsFlexible matches CDR flexibility in MD simulations

Although crystallographic data can reveal the conformational states of CDRs, it does not directly measure flexibility, and there always remains the possibility that additional conformational states exist but have not been captured. A limitation of the ALL-conformation dataset, therefore, is the lower confidence of rigid compared with flexible labels. Although we introduced the requirements to address this limitation (Methods), the true extent of flexibility may well be underestimated.

**Fig. 4:**
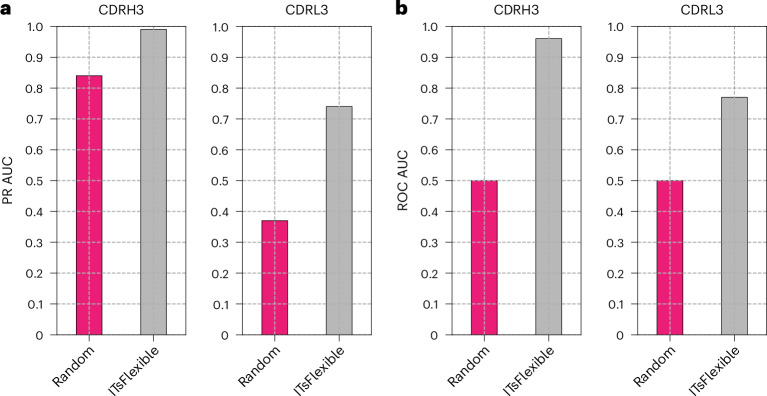
ITsFlexible performance evaluated on the MD test set containing 19 antibodies. **a**,**b**, Classification of CDRH3s and CDRL3s is evaluated with metrics of PR AUC (**a**) and ROC AUC (**b**). A prediction was made for each representative structure extracted from MD and classification performed based on the maximum ITsFlexible score observed across the ensemble. Exact PR AUC and ROC AUC values and performance based on the mean ITsFlexible score observed across the ensemble are presented in Supplementary Table 17.

We used MD simulations to classify CDR flexibility by simulating a set of 19 antibodies and labelling the flexibility of CDR3s (Supplementary Table 2). As expected, we observed a higher proportion of flexible CDR3s in MD (84% for CDRH3 and 37% for CDRL3) than the crystal structure data (64% for CDRH3 and 18% for CDRL3). ITsFlexible achieved near-perfect separation for CDRH3s, with slightly worse performance for CDRL3s (Fig. 4), suggesting that ITsFlexible also detects signals promoting flexibility in physics-based molecular simulations.

#### Cryo-EM experiments confirm predicted flexibility

We chose three antibodies as challenging test cases for experimental validation using cryo-EM. Antibodies in the patent and literature antibody database^49^ against the influenza H1N1 hemagglutinin (HA) with ITsFlexible score below 0.1 (rigid examples) or above 0.5 (flexible examples) were considered. Three antibodies with low sequence identity to any examples in the training set and loop lengths opposing trends observed in ALL-conformations (long loop for rigid case and shorter loops for flexible cases) were selected. These were imaged in complex with the antigen and the heterogeneity in the density maps, indicative of conformational variety^50^, was analysed.

**Fig. 5:**
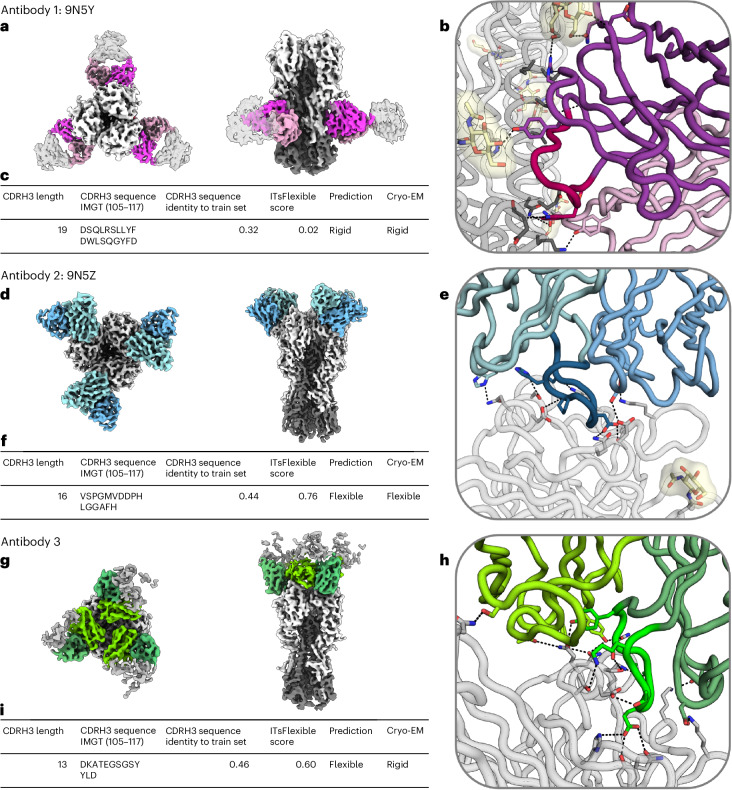
Case-study antibodies selected for cryo-EM experiments. High-resolution structure of the three antibodies in complex with influenza H1N1 HA were solved. **a**,**d**,**g**, Top and side views of the cryo-EM map of antibodies (heavy chain, dark colour; light chain, bright colour) in complex with the antigen (grey). In each structure, three symmetrically arranged copies of the antibody were captured. Antibody 1 (9N5Y; **a**) binds to the HA stem and antibodies 2 (9N5Z; **d**) and 3 (**g**) to the HA head. **b**,**e**,**h**, Cartoon representation of antibody–antigen binding interfaces of antibodies 9N5Y (**b**), 9N5Z (**e**) and 3 (**h**). CDRH3s are shown in different shades of colour and binding interactions are highlighted in stick representation. **c**,**f**,**i**, Summary tables showing the CDRH3 length and sequence, sequence identity to the closest example in the ITsFlexible training set, the ITsFlexible prediction score (a higher value indicates a higher likelihood of being flexible), a binary prediction of loop flexibility based on the ITsFlexible score and the flexibility determined with the cryo-EM experiments for antibodies 9N5Y (**c**), 9N5Z (**f**) and 3 (**i**). Additional metadata for the three antibodies are shown in Supplementary Table 21.

Antibody 1 is as an example of a long CDRH3 (length 19, longer than 86% of CDRH3s in ALL-conformation) predicted to be rigid with high confidence (ITsFlexible score of 0.02). The cryo-EM data revealed that the majority of good particles selected from two-dimensional classification adopt one homogeneous three-dimensional (3D) class, which was used to build a high-resolution consensus structure (Fig. 5). In agreement with model prediction, these data show that the CDR occupies a single conformation.

Antibody 2 is an example with a slightly shorter CDRH3 loop (length 16) predicted to be flexible with high confidence (ITsFlexible score of 0.76). Our initial data processing showed high flexibility in the binding interface of the antibody and the antigen although at a low resolution. With further 3D classification and data collection, we were able to obtain a high-resolution structure of one of the binding states (Fig. 5). Data resolution did not allow us to build models of distinct conformational states, which makes it hard to localize the flexibility to the CDRH3 loop. However, the density maps show clear conformational heterogeneity at the binding interface and induced by the antibody (Supplementary Videos 1 and 2). Flexibility of CDRH3 is a likely cause for this observation.

Antibody 3 is a further example of a CDRH3 predicted to be flexible. The CDRH3 is even shorter than in antibody 2 (length 13) and the ITsFlexible score of 0.60 suggests a lower prediction confidence. The cryo-EM data disagreed with the prediction and revealed no heterogeneity (Fig. 5).

A potential limitation of the experimental setup is that antibodies were imaged in complex with the antigen. This choice was made as the minimum particle size required for cryo-EM complicates the study of free antibodies. Binding can rigidify residues as additional molecular interactions with the antigen impose extra constraints^7^, and we expect ITsFlexible predictions to better match the flexibility of the unbound antibody (see the ‘Discussion’ section). This limitation could be a reason for the absence of flexibility observed in experiments for antibody 3. In particular, we observe that a glutamate residue at the tip of the CDRH3 forms three hydrogen-bond interactions with the antigen (Fig. 5). Accordingly, an argument could also be made that the unbound state of antibody 1 is more flexible than observed. The data, nevertheless, provide clear evidence for reduced flexibility of antibody 1 compared with antibody 2 when imaged under the same conditions, matching our predictions.

### Discussion

Conformational changes are fundamental to the functional properties of many proteins^1,2^. In antibodies and TCRs, flexibility—especially of the highly important CDR3s—impacts key properties such as affinity and specificity ^5–8^. However, predicting protein flexibility remains challenging, as current structure prediction tools struggle to capture multiple conformational states, particularly for antigen receptor CDRs^21,36^. One factor that has limited the progress in conformation prediction is the absence of large datasets necessary to train and evaluate such machine learning models.

Here we focused on the computational prediction of antigen receptor CDR3 flexibility. To help overcome the lack of data, we collected structural motifs with the same secondary structure pattern, defined as a loop connecting two consecutive antiparallel β-strands, across all proteins. Mining the PDB, we created ALL-conformations—a dataset containing more than 1.2 million crystal structures of such loops with more than 100,000 unique sequences. Analysing the conformational flexibility in ALL-conformations, we were able to label 20,000 loops with high confidence as either flexible (adopting multiple conformations) or rigid (occupying a single state).

Using the ALL-conformations dataset, we developed ITsFlexible—a method for the classification of CDR loops as either flexible or rigid. The model was trained on the subset of loop motifs from general proteins in the PDB and evaluated on its ability to predict the CDR flexibility in an out-of-distribution setting. ITsFlexible achieved state-of-the-art performance on crystal structure test sets and generalized effectively to the MD simulation data. In addition, we showed that the model achieves similar performance when using inputs of structural models rather than crystal structures, highlighting its applicability to antibodies without experimentally solved structures.

An ablation of ITsFlexible inputs indicated biophysical factors that influence the flexibility of CDRs. In line with previous MD studies^44^, we identified the arrangement of residues in the structural context of CDRs as a key factor driving flexibility.

Furthermore, our analysis showed that uncertainty scores of protein structure prediction tools and diversity in structural ensembles generated with established workflows are not reliable predictors of CDR flexibility. The AF2 pLDDT score has been described as a good indicator for disordered regions of proteins^13^; however, we found that it was not highly predictive of CDR flexibility. Similarly, the predicted error of the antibody-specific ABB2 correlates more strongly with the number of times a particular sequence was present in the training set than flexibility. We also assessed the diversity of CDR ensembles produced by AF2 MSA subsampling as a predictor of flexibility. MSA subsampling is a popular workflow to model multiple conformational states of proteins^22,23,26–30^. Although this approach is more predictive than confidence scores, the workflow again does not accurately capture CDR flexibility.

We experimentally evaluated ITsFlexible’s predictive performance using three antibodies chosen as challenging test cases. The antibodies had low sequence identity to the training set and the loop length opposed trends observed in the data (long loops for rigid cases and shorter loops for flexible cases). Using cryo-EM, we imaged the antibodies in complex with their antigen and analysed heterogeneity in the density maps^50,51^. For two of the three cases, the experimental evidence confirmed our predictions. Among the correctly predicted cases, one rigid and one flexible, the former was three residues longer, further demonstrating that the model detects features beyond loop length associated with flexibility. The third antibody featured an even shorter CDRH3 predicted to be flexible, although with lower confidence, and experiments showed no evidence of conformational heterogeneity. Overall, cryo-EM experiments provide strong evidence that ITsFlexible is predictive of real CDR3 conformational dynamics observed in solution.

One limitation of this work is the approach of identifying flexibility. We label a CDR as flexible if we observe multiple conformational states irrespective of whether the antigen receptor is bound to the antigen or in its free state. Previous studies showed that the bound conformation is frequently included in the unbound ensemble, suggesting that conformational selection is a more common mechanism of antibody binding than induced fit^4,52,53^. We, therefore, expect that ITsFlexible predictions are representative of the flexibility of unbound CDRs. This assumption is supported by the agreement with MD simulations of unbound antigen receptors that we observe. As some functional properties (such as binding affinity) are dependent on the balance of flexibility in bound and unbound states^7^, we suggest the prediction of changes in CDR flexibility on binding as an important future direction. Owing to the limited amount of data capturing CDR conformational states, however, predicting flexibility conditional on the antigen is currently challenging.

In this work, we present ALL-conformations^41^ and ITsFlexible (available at https://github.com/oxpig/ITsFlexible and in ref. ^42^). ALL-conformations captures the full range of experimentally observed conformational diversity of loops between antiparallel β-strands, enabling a detailed analysis of loop dynamics and supporting the training and benchmarking of more robust conformation prediction workflows. ITsFlexible accurately predicts the CDR conformational flexibility and is able to help with key problems in drug design. CDR flexibility has been linked to reduced binding affinity^7,8^ and increased polyspecificity^5,6^, both of which are typically undesirable when engineering antigen receptors for therapeutic use^10^. By allowing the rapid screening of candidate molecules, ITsFlexible can help identify receptors with more favourable therapeutic profiles. Moreover, flexibility predictions can help identify antigen receptors for which computationally expensive MD simulations are likely to yield the greatest benefit and support a more strategic use of computational resources. Several studies have shown that incorporating structural ensembles of flexible molecules can improve downstream tasks, such as antibody–antigen docking^54,55^. Taken together, the development of ALL-conformations and ITsFlexible paves the way for tackling more complex tasks such as sampling structures of different conformational states in the future.

## Methods

### ALL-conformations dataset

CDR3s of proteins in the immunoglobulin superfamily share a common secondary structure; they are formed by a loop that connects two antiparallel β-strands (Supplementary Fig. 4). We created ALL-conformations—a dataset consisting of five subsets that captures all the observed conformational states of antibody CDRH3s and CDRL3s, TCR CDRB3s and CDRA3s and loop motifs between antiparallel β-strands across all proteins in the PDB. To generate the datasets, we implemented a systematic approach to search protein structure databases (PDB^38^, SAbDab^39^ and structural T cell receptor database^40^) for all the solved structures of the five protein motifs.

#### The PDB set

We mined all the protein structures deposited in the PDB before 22 November 2023 (ref. ^38^) for loop motifs sitting between two adjacent antiparallel β-strands. We used the define secondary structure of proteins algorithm^56^ to assign the secondary structure to amino acid residues. We then identified all the antiparallel β-strands labelled by the algorithm and extracted the regions (loops) in between the two strands. The obtained loop structures were quality filtered for those solved by X-ray crystallography with resolution under 3.5 Å and no unresolved loop residues. A maximum of three residues forming secondary structure elements of β-strand or α-helix within each loop were allowed.

#### Antibody and TCR CDR3 sets

All the antibody fragment variable (Fv) structures were extracted from SAbDab^39,57^. Both standard Fvs and single-chain Fvs were included. All TCR Fv structures were extracted from the structural T cell receptor database^40^. Multiple copies of antibodies in the same PDB structure were extracted as CDRs can adopt distinct conformations. Furthermore, some structures contain residues with alternate atom coordinates. These states were separated and individually added to the dataset. Structures were filtered for those solved by X-ray crystallography with a resolution below 3.5 Å, presence of a complete Fab (both heavy and light chains present in the antibody structures, and α- and β-chains or γ- and δ-chains for TCRs) and no unresolved residues in any of the CDRs.

Here we defined CDR3 loops as the IMGT-numbered^58^ residues 107–116, which differ from the standard definitions. This choice was made to ensure that the definition of a CDR3 is more consistent with the way loops are defined by their secondary structure in the PDB set. The standard definition of a CDR3 loop in the IMGT-numbering scheme are residues 105–117. However, an analysis of all antibody structures in SAbDab^39^ showed that the residues at the start and end of IMGT definitions tend to be part of β-strands on either side of the loop (Supplementary Fig. 4). Positions 107–116 correspond better with the residues that sit between the two β-strands, and therefore, we used this range to define CDR3s throughout this paper.

#### Conformational flexibility

We grouped the structures in ALL-conformations into sets of loops we consider identical. For the PDB set, we grouped structures by sequence identity of the loop and considered all structures within a group to depict the same loop irrespective of the sequence of the rest of the protein. Antibody and TCR structures were grouped by sequence identity of the entire Fv rather than simply the CDR3s. In this way, CDR3s are only considered to be the same if the entire domain is identical.

For loops with multiple available structures, we analysed the conformational flexibility by calculating the number of accessible conformations. We defined a conformation based on the structural similarity of a loop, using the RMSD of Cα atoms. We clustered sequence-identical loops using an agglomerate clustering algorithm with complete linkage^59^ and 1.25-Å distance threshold. This enforces that any two structures within a conformation have a maximum RMSD of 1.25 Å. This clustering approach was chosen as it is known to provide a good functional clustering of CDR loops in antibodies^43^. The structural similarity of loops can be calculated in multiple ways (Supplementary Section 2.2.2 shows another choice).

Each loop was assigned a label of flexible, rigid or ‘unknown’. Loops for which multiple conformations (clusters) were found were assigned the flexible label. Loops for which only a single conformation was observed were not automatically assigned the rigid label. The absence of multiple observed conformations does not prove that a loop cannot adopt multiple conformations. It is possible that alternative states were not captured by the limited number of crystal structures available. The more structures that are available of a loop in which it adopts a single conformation, the more confident we can be on the absence of flexibility. Therefore, a requirement was introduced that a loop needs to adopt a single conformation in at least five separate PDB files to be labelled as rigid. We chose the requirement of five separate PDB files instead of simply five occurrences as the same loop can occur several times in the same PDB file due to multiple copies of a protein within a crystal unit cell. Loops neither labelled as flexible or rigid were assigned to the unknown group.

### ITsFlexible

ITsFlexible is a binary classifier. It takes as input a structural representation of a CDR loop motif and classifies it as flexible (able to adopt multiple conformations) or rigid (occupies a single stable conformation). The model architecture and training procedure are outlined below and details are provided in Supplementary Information. The model is available via GitHub (https://github.com/oxpig/ITsFlexible) and Zenodo^42^.

#### Model architecture

The ITsFlexible model is a graph neural network (Supplementary Fig. 1) and consists of three equivariant graph convolutional layers^60^. The layers take input of node features, coordinates and edge features. Node features are iteratively updated and the last layer of node embeddings are pooled. A linear layer with the sigmoid activation function is applied for binary classification. The chosen model architecture makes predictions invariant to transformations of the group E(3) (translations, rotations and reflections); therefore, orientations and absolute positions of the input protein structure can be ignored. Predictions are dependent only on the relative residue distances.

#### Model inputs

A loop and its structural context were encoded as a residue-level graph. The context was provided by all residues within 10 Å of any loop residues. For simplicity in the PDB set, only residues located on the same protein chain as the loop were selected as context. For antibodies and TCRs, residues located on both immunoglobulin chains were included, as both chains influence conformational flexibility^44^. Node features were a 22-dimensional vector consisting of a one-hot encoding of amino acid type (1 class for each of the 20 amino acids plus an additional class for unknown residues) and a one-hot encoding (1 class) whether the residue is located in the loop or structural context. Non-standard amino acids closely related to a standard amino acid were encoded as such; others were encoded as unknown residues (Supplementary Table 1). The amino acid encoding was concatenated with a one-hot encoding of the residue being located in the loop or the context. Coordinates for each node were taken as the position of the Cα atom of a residue. Nodes were locally connected with edges using a 10-Å distance threshold. Edge features are nine dimensional, providing a one-hot encoding of the presence of a covalent bond between two residues and Cα distance encoding. The distance encoding was produced by eight Gaussian radial basis functions equally distributed between 0 Å and 10 Å.

#### Training

ITsFlexible was trained on the ALL-conformations PDB set using a 70–15–15 training, validation and test split. For length-matched loops, a maximum sequence identity of 80% was allowed between the splits. Additionally, all loops with more than 80% aligned sequence identity to any loop (not restricted to matching loop length) in the ALL-conformations CDRH/L/A/B3 sets were removed from the training and validation sets. For the training set, we sampled five structures per loop randomly to ensure the stability of predictions to small changes in atom coordinates. For the validation set, one structure per loop was sampled at random.

ITsFlexible was trained with a binary cross-entropy loss using the Adam optimizer^61^ with a learning rate of 2 × 10^−4^ and a weight decay of 10^−6^. During training, edges were dropped at random with a probability of 0.2. The PR AUC value was monitored and training was stopped when converged. Ten models were trained and the one with the best validation PR AUC value was selected. All models were trained on an NVIDIA Quadro RTX 6000 GPU, in approximately two GPU hours per model.

#### Evaluation

ITsFlexible performance was evaluated using structural inputs derived from crystal structures and predicted structural models. When evaluating crystal structures, we made an ITsFlexible prediction for each available experimental structure containing a loop and took the mean prediction score to calculate the performance metrics. To evaluate ITsFlexible with input derived from the structural models, we predicted antibody and TCR structures with IB and AF2. IB predictions are made from paired Fv sequences using the ABB2 (for antibodies) and TCRBuilder2 (for TCRs) models with default parameters. AF2 predictions of antibodies and TCRs were made using the ColabFold implementation of AlphaFold-Multimer^62^ with default parameters.

### Baseline models

A set of three baseline models were created to classify loop flexibility based on simple biophysical input features. The first baseline was formed by a logistic regression classifier fit to inputs of loop length. Longer loops contain more bonds around which they can rotate and are, therefore, expected to be more flexible in conformation than shorter ones^20,44^. We found a moderate correlation between loop length and flexibility in our datasets (Supplementary Table 4). The second baseline was a logistic regression classifier fit to inputs of the solvent exposure of a loop. We approximated solvent exposure by the number of residues located within a 10-Å radius around the loop. Loops with higher solvent exposure have less steric hindrance restricting conformational rearrangements and are expected to be more flexible^44^. A final baseline model was formed by a logistic regression classifier fit to inputs of both length and solvent exposure. All the baseline models were fit on the training split of the PDB set.

### Alternative flexibility classifiers

Three alternative workflows were implemented to predict CDR flexibility. These were based on protocols designed to model protein conformational ensembles and confidence metrics of protein structure prediction tools.

#### AF2 pLDDT

AF2 returns pLDDT scores for predicted structures, which can be interpreted as a residue-level confidence measure of the prediction. Antibody and TCR structures were predicted using the ColabFold implementation of AlphaFold-Multimer^62^ with default parameters. The pLDDT score of the highest-ranked model was extracted. The mean pLDDT of residues located in a CDR was used as the input of a logistic regression model to classify flexibility.

#### AF2 MSA subsampling

An AF2 MSA subsampling workflow, based on an established protocol^21,22^, was used to predict the structural ensembles. AlphaFold-Multimer^63^ was run using the ColabFold^62^ implementation to model antibody and TCR structures. We set the maximum MSA depth to 64, extra sequences to 128 and the number of recycles to 1 (default 3 in AF2). Low values of all three parameters increase the diversity of predicted structures and improves the sampling of alternative conformations. Setting parameters too low can lead to the generation of unfolded structures^21^. We chose these parameter values as they are within ranges described in previous studies^21,22^ and they led to the maximum diversity and limited the occurrence of unfolded structures for the antibodies in our test set. ColabFold was run with 8 seeds resulting in 40 models (5 models are produced per seed) for each protein. The MSA subsampling protocol took approximately 3 min on a NVIDIA A100 GPU. Owing to the computational cost, MSA subsampling was only performed for the smaller CDR sets and not the full PDB set.

We used the magnitude of structural diversity observed across the 40 predicted structures as a zero-shot classifier of CDR flexibility. Structural diversity was calculated by the Cα RMSD of the CDR loop residues. The mean structural diversity was used as the input of a logistic regression model to classify flexibility.

#### ABB2 RMSPE

The ABB2-predicted error, a residue-level confidence metric^45^, was used to classify the flexibility of antibody CDRs. Antibody structures were predicted using a retrained version of ABB2. There is a large overlap of antibodies in the CDR test set used here for evaluation of flexibility prediction and the training set of ABB2. To avoid data leakage, we retrained ABB2, removing all antibodies with 100% CDRH3 or CDRL3 sequence identity to any CDR in the test sets. This reduced the training set to 4,469 antibodies compared with 5,669 antibodies in the original training set. This version of ABB2 retains good accuracy at antibody structure prediction evaluated on a benchmark (Supplementary Table 19). For details on the ABB2 retraining as well as an evaluation of the original ABB2 for flexibility prediction, see Supplementary Section 2.4.

Antibody Fv structures were predicted using ABB2 with the retrained weights. For all the remaining parameters, ABB2 default values were used. The predicted error of modelled antibodies was extracted. The RMSPE of CDR residues was used as the input of a logistic regression model to classify flexibility.

### MD simulations

MD simulations were performed following the protocol described in ref. ^64^. The 19 investigated antibodies (Supplementary Table 2) were prepared at pH 7.4 using the Protonate3D tool in MOE^65,66^. C-termini were capped with N-methyl groups. Each structure was solvated in a cubic TIP3P water box with a minimum wall distance of 12 Å from the protein^67–69^, and all systems were parameterized using the AMBER ff19SB force field. A uniform background charge was applied to neutralize the system and enable an accurate calculation of long-range electrostatics^70^.

To broaden the exploration of conformational space, we first performed enhanced sampling using well-tempered metadynamics, implemented in GROMACS with PLUMED 2 (refs. ^71–76^). The collective variables were defined as a linear combination of the sine and cosine of the *ψ* torsion angles of either the CDRL3 and CDRH3 loops or all the CDR loops. Well-tempered metadynamics simulations were run for 1,000 ns per system, with a Gaussian height of 10 kJ mol^−1^, a deposition interval of 5,000 steps and a bias factor of 10. To mitigate/reduce any bias introduced by enhanced sampling and to obtain trajectories representing unbiased dynamics, we used a two-step procedure. First, the well-tempered metadynamics trajectories were aligned on the Cα atoms of the variable domains and clustered on the CDR loops using average linkage hierarchical clustering (RMSD cut-off of 1.2 Å) in CPPTRAJ^77^. Representative cluster structures were used as the starting points for independent unbiased MD simulations (100 ns per replicate), providing an aggregated sampling of >10 μs per system.

Classical MD simulations were carried out in the AMBER 22 simulation package^78^, using the AMBER ff19SB force field and TIP3P solvent. Simulations were performed in an *NpT* ensemble using pmemd.cuda^79^, with temperature maintained at 300 K via Langevin dynamics (collision frequency, 2 ps^−1^)^80,81^ and pressure maintained with a Monte Carlo barostat (one volume change per 100 steps)^82^. Bonds involving hydrogen atoms were constrained with SHAKE, enabling a 2-fs time step.

To extract representative conformations, we performed hierarchical clustering of the unbiased MD trajectories using average linkage clustering (for RMSD cut-off of 2.5 Å, the CDR loops aligned on the Fv domains), as implemented in CPPTRAJ.

To determine binary labels of flexible and rigid for the CDRs of the simulated antibodies, we performed analysis identical to the crystal structure datasets (see the ‘ALL-conformations dataset’ section). The representative MD structures were clustered by Cα RMSD of CDR3 loops using an agglomerative clustering algorithm with complete linkage^59^ and a 1.25-Å distance cut-off. The number of conformations observed for the CDR3s of each antibody is listed in Supplementary Table 2. CDRH3s and CDRL3s were then assigned the labels of flexible (if multiple conformations were observed in the simulations) or rigid (in case of a single conformation).

The flexibility of simulated antibodies was predicted by ITsFlexible. A prediction was made for each representative structure of an antibody and the maximum prediction score was used. We also tried using the mean prediction score. This, however, resulted in a more narrow distribution of scores across the dataset, which is less optimal for classification tasks.

### Cryo-EM experimental protocol and data analysis

The patent and literature antibody database^49^ was searched for antibodies that target influenza H1N1 HA. Antibody Fv structures were predicted with a default run of ABB2 (ref. ^45^). Flexibility was classified with ITsFlexible from inputs of the obtained structural models. Antibodies with a predicted probability score lower than 0.1 were considered as rigid examples and antibodies with a score above 0.5 as flexible examples. Three antibodies (one rigid and two flexible) with good developability properties predicted with therapeutic antibody profiling^83^ were selected for the cryo-EM experiments.

#### Sample preparation

Three HA (HA/California/07/2009) in complex with one Fab each (AEL31302/AEL31311 (antibody 1), AMB38310/AMB38599 (antibody 2) and AMB38442/AMB38568 (antibody 3)) were prepared by mixing Fab with a 1:1 molar ratio of Fab:HA protomer and incubated for approximately 4 h at 4 °C. Also, 0.25 μl of 4-mM CHAPSO detergent was added to the complex to aid in particle tumbling. The corresponding final concentration for the complexes of HA with each Fab were 1.14 mg ml^−1^, 1.11 mg ml^−1^ and 1.02 mg ml^−1^ on the grid. The samples were added to glow-discharged 1.2/1.3 UltrAuFoil 300-mesh grids and subjected to vitrification using a Vitrobot Mark IV system. The settings were as follows: 3 μl of sample, temperature inside the chamber was 4 °C, humidity was 90%, blotting force was 1 and wait time was 3 s. The sample was blotted off for 4.5 s and the grids were plunge frozen into liquid-nitrogen-cooled liquid ethane.

#### Data collection, processing and model building

Cryo-grids of the complexes were imaged at ×190,000 nominal magnification using a Falcon 4i camera on a Glacios microscope at 200 kV. Automated image collection was performed using EPU from Thermo Fisher. Micrographs for antibody 1 were collected untilted and micrographs for antibody 2 were collected with a 30° tilt. Images were aligned, dose weighted and contrast transfer function corrected in the CryoSPARC Live software platform, with automated image collection also performed using Smart EPU software (Thermo Fisher). Data processing for all three datasets was carried out in CryoSPARC (v. 4.5.3)^84^. Blob particle picking was performed on all micrographs with a minimum particle diameter of 100 Å and a maximum particle diameter of 200 Å. Particles extracted at 480-pixel box size were used to perform two-dimensional classification for antibody 1 and antibody 2, which were then used to generate a 3D reference model from ab initio refinement, followed by heterogeneous refinement to obtain one good class that was further non-uniform heterogeneous refined. For antibody 3, we extracted particles at 512 pixels and Fourier cropped to 256-pixel box size to perform two-dimensional classification, followed by ab initio, heterogenous and non-uniform refinements. gold-standard Fourier shell correlation resolution was calculated to be 3.77 Å for antibody 1 and 3.91 Å for antibody 3. We docked the models into the cryo-EM density map in UCSF ChimeraX^85^. The structure model was built iteratively with Coot followed by real-space refinement in the PHENIX package^86^. The Kabat numbering system^87^ was used for antibodies and H3 numbering scheme for HA.

For antibody 2, initially, no high-resolution map could be obtained as the antibody reveals a high degree of variability contributing to conformational changes in the head and stem of HA. The workflow to visualize the conformational changes in the complex is shown in Supplementary Fig. 2. We used 243,000 particles and the map resulting from the non-uniform refining job downsized to 128 pixels as input for the 3D flex^50^ data preparation job. For the 3D mesh prep job, a solvent mask was generated by low-pass filtering the consensus map from the 3D flex data prep job and segmented as shown in Supplementary Fig. 2. A base number of 40 tetrahedral cells was used in combination with a minimum rigidity weight of 0.5. The resulting 3D flex mesh was used to run a 3D flex train with a number of latent dimensions of 3. A 3D flex generator was used to produce a volume series of 41 frames using the consensus map, showing the areas of high flexibility. A video depicting the conformational flexibility based on the volume series was generated using UCSF ChimeraX^85^.

### Reporting summary

Further information on research design is available in the Nature Portfolio Reporting Summary linked to this article.

## Supplementary information

Supplementary Information Supplementary Sections 1 and 2, Figs. 1–11, Tables 1–21, Methods, and Results.

Reporting Summary

Supplementary Video 1 Conformational flexibility of antibody 9N5Z prepared with workflow 1.

Supplementary Video 2 Conformational flexibility of antibody 9N5Z prepared with workflow 2.

## Data Availability

The ALL-conformations dataset and representative structures of MD-simulated antibodies are available via Zenodo (10.5281/zenodo.15784241)^41^. High-resolution cryo-EM structures of antibody 1 and antibody 2 are deposited in the PDB under codes 9N5Y and 9N5Z, respectively.

## Code availability

ITsFlexible method is publicly available via GitHub (https://github.com/oxpig/ITsFlexible) and Zenodo (10.5281/zenodo.16891380)^42^.
