# Supplementary material for: Predicting the conformational flexibility of antibody and T cell receptor complementarity-determining regions
Source: Nat Mach Intell. 2025 Oct 16;7(10):1755–67. doi: 10.1038/s42256-025-01131-6 (PMC12552124; doi:10.1038/s42256-025-01131-6)
Supplement: Supplementary file 1 — Supplementary Sections 1 and 2, Figs. 1–11, Tables 1–21, Methods, and Results. [file 42256_2025_1131_MOESM1_ESM.pdf]

nature machine intelligence

Supplementary information https://doi.org/10.1038/s42256-025-01131-6

Predicting the conformational flexibility of antibody and T cell receptor complementarity-determining regions

In the format provided by the authors and unedited

Contents

S1 Supplementary methods **2**

S1.1 ITsFlexible models . . . 2

S1.1.1 ITsFlexible . . . 2

S1.1.2 ITsFlexible-Loop . . . 4

S1.1.3 ITsFlexible-Sequence . . . 4

S1.2 Amino acid encoding . . . 5

S1.3 Molecular dynamics dataset . . . 5

S1.4 Cryo-EM . . . 5

S2 Supplementary results **9**

S2.1 CDR3 secondary structure . . . 9

S2.2 ALL-conformations . . . 9

S2.2.1 ALL-conformations statistics . . . 9

S2.2.2 Alternative definition of loop flexibility . . . 11

S2.3 Extended evaluation of ITsFlexible performance . . . 12

S2.3.1 Consistency of predictions from multiple structures of the same loop . . . 12

S2.3.2 Predictions from structural models . . . 13

S2.3.3 Performance with secondary structure-based CDR3 definitions . . . 13

S2.3.4 Performance with alternative definition of flexibility . . . 14

S2.3.5 Impact of varying RMSD thresholds on datasets and model performance . 15

S2.3.6 Supplementary tables . . . 18

S2.4 ABodyBuilder2 . . . 22

S2.4.1 ABB2 retraining . . . 22

S2.4.2 Flexibility classification with ABB2 . . . 22

S2.5 Case study antibodies for cryo-EM . . . 25

S1 Supplementary methods

S1.1 ITsFlexible models

S1.1.1 ITsFlexible

ITsFlexible is an equivariant graph neural network (EGNN). The model takes a geometric graph as input that contains node features, coordinates and edge features and outputs a probability score that indicates the probability of a data point belonging to the positive class (overview in Figure S1 and details in Algorithm 1). Initially, node features are embedded into a 128 dimensional vector. Node embeddings are then updated through 3 E(n)-equivariant graph convolutional layers (EGCLs) (Satorras et al., 2022):

$$
\begin{aligned}
m_{ij} &= \phi_m(h_i^l, h_j^l, \|x_i^l - x_j^l\|^2, a_{ij}) \\
x_i^{l+1} &= x_i^l + C \sum_{j \neq i} (x_i^l - x_j^l)\ \phi_x(m_{ij}) \\
m_i &= \sum_{j \neq i} m_{ij} \\
h_i^{l+1} &= \phi_h(h_i^l, m_i)
\end{aligned}
$$

where h^l are the node embeddings, x^l are the coordinate and a_{ij} the edge features. The last layer of node embeddings are maxpooled and a linear layer with sigmoid activation function is applied for binary classification. Coordinate updates in the EGCL are equivariant with respect to the group E(3) and node embedding updates are invariant. Classification of ITsFlexible is therefore invariant to translations and rotations of the input structure.

Algorithm 1 ITsFlexible

Require: Node features $n \in \mathbb{R}^{N_{nodes} \times 22}$, node coordinates $x \in \mathbb{R}^{N_{nodes} \times 3}$, edges $a \in \mathbb{R}^{N_{edges} \times 9}$

def ITsFlexible(n, x, a)
  $h_n^0 \leftarrow \text{Linear}(n_n)$ $h_n \in \mathbb{R}^{128}, n \in \mathbb{R} \cap [0, N_{nodes}]$
  for $l = 1$ **to** 3
    $a^l = \text{DropoutEdge}(a)$
    $h^l, x^l = \text{EGCL}(h^{l-1}, x^{l-1}, a^l)$
    $h^l = h^l + h^{l-1}$
  end for
  $h^4 = \text{MaxPool}(h^3)$ $h^4 \in \mathbb{R}^{128}$
  $o = \text{Sigmoid}(\text{Linear}(h^4))$ $o \in \mathbb{R} \cap [0, 1]$
  return o

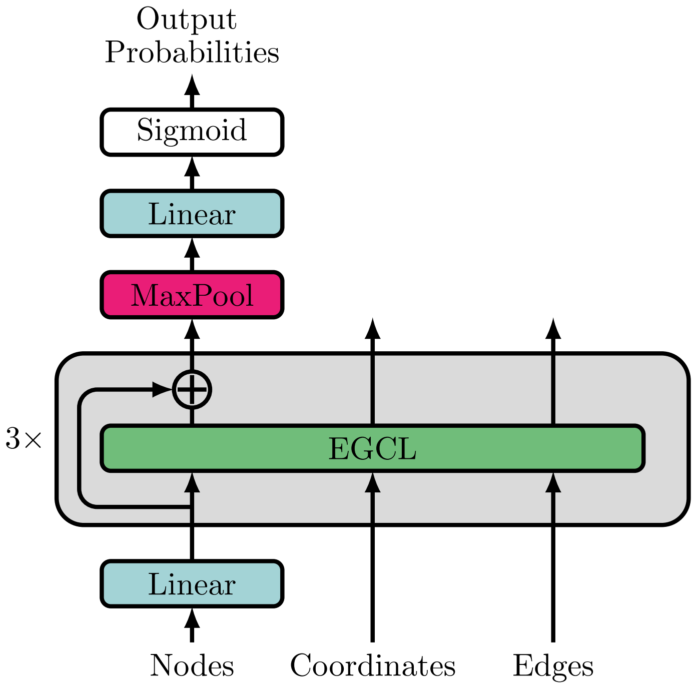

Figure S1: ITsFlexible model architecture. ITsFlexible is a GNN that consists of 3 E(n)-equivariant graph convolutional layers (EGCLs). The final node features are max pooled and a classification head with linear layer and sigmoid activation is applied.

S1.1.2 ITsFlexible-Loop

ITsFlexible-Loop is an EGNN very similar in architecture and training to ITsFlexible. The main difference is the model input. The ITsFlexible-Loop input is a graph encoding of only loop residues, the structural context is not provided. The coordinates and edge features of the graph are generated as described in the methods section in the main of this manuscript. As all nodes are located in the loop, node features are reduced to a 21-dimensional one-hot encoding of amino acid type. The remaining hyperparameters are the same as for the ITsFlexible model.

S1.1.3 ITsFlexible-Sequence

ITsFlexible-Sequence is a CNN-based model which classifies loops from inputs of a sequence representation. Algorithm 2 shows details of the model. The model input is a one-hot encoding of the amino acid sequence. Each residue is encoded as 21 dimensional vector (1 class for each of the 20 amino acids plus an additional class for unknown residues). Non-standard amino acids closely related to a standard amino acid are encoded as such, others are encoded as unknown residues (Table S1). Encodings are padded with zeros to a standard length of 51 residues. This enforces a length limit of 51 residues for input sequences, which corresponds to the longest loop observed in the PDB (Berman, 2000). A sequence embedding is produced with two 1D-convolutional blocks with 256 channels. The final embedding is flattened to a vector of dimensions 51×256. Two linear layers followed by a sigmoid activation function are used for binary classification from the sequence embedding.

ItsFlexible-Sequence was trained on the PDB set with identical splits as ITsFlexible (see methods). ITsFlexible-Sequence was trained with a binary cross-entropy loss using the Adam optimiser (Kingma & Ba, 2014) with a learning rate of $2 \cdot 10^{-4}$. During training dropout of 0.2 was used in the convolutional layers and dropout of 0.05 in the linear layers. The validation area under the precision-recall curve (PR-AUC) was monitored and training stopped when converged. Ten models were trained and the one with the best validation PR-AUC selected.

Algorithm 2 ITsFlexible-Sequence

Require: Loop sequence encoding $s \in \mathbb{R}^{N_{residues} \times 21}$

```
def ITsFlexibleSequence(s)
   i = ZeroPad(s)                                   i ∈ R^{51×21}
   h^0_n ← i_n                                      n ∈ R ∩ [0, 51]
   for l = 1 to 2
      h^l ← ReLu(Conv1d(h^{l−1}))                   h^l ∈ R^{51×256}
      h^l = Dropout(h^l)
      h^l = MaxPool1d(h^l)
   end for
   h^3 ← Flatten(h^2)                               h^3 ∈ R^{(51×256)}
   h^4 ← Dropout(ReLu(Linear(h^3)))                 h^4 ∈ R^{512}
   o ← Sigmoid(Linear(h^4))                         o ∈ R ∩ [0, 1]
   return o
```

S1.2 Amino acid encoding

When encoding ITsFlexible inputs, non-standard amino acids were represented either as a unknown residue or as the closest related standard amino acid. Non-standard amino acids that occur frequently in the test sets and are closely related to one of the 20 standard amino acids were encoded as such. Table S1 shows a map of the selected non-standard amino acids and their encoding.

Table S1: Encoding of non-standard amino acids

Non-standard amino acid	Encoded as
Beta-L-aspartic acid (IAS)	Aspartic acid (ASP)
S-hydroxycystein (CSO)	Cystein (CYS)
4-methyl-histidine (HIC)	Histidine (HIS)
N-dimethyl-lysine (MLY)	Lysine (LYS)
Selenomethionine (MSE)	Methionine (MET)
Phosphoserine (SEP)	Serine (SER)
Topaquinone (TPQ)	Tyrosine (TYR)
3-amino-6-hydroxy-tyrosine (TYQ)	Tyrosine (TYR)

S1.3 Molecular dynamics dataset

19 antibodies were simulated with our molecular dynamics protocol described in the methods section of this manuscript. The number of conformations observed for CDRH3s and CDRL3s is shown in Table S2.

Table S2: Number of conformations observed in molecular dynamics simulations

Antibody	N CDRH3 conformations	N CDRL3 conformations
1BFO	3	1
1JPS	3	1
1MLB D44	3	2
2Q76 D44	1	1
2VXT	1	2
2Y06	3	1
2Y07	3	1
2Y36	3	1
3EOA	5	1
3G6D	3	4
3HI6	4	2
3RVW	6	2
3V6F	9	6
4KMT	2	1
5I15	6	1
5I18	9	1
5I1A	3	1
7G12	3	2
7G12 mature	1	1

S1.4 Cryo-EM

Table S3: Cryo-EM data collection, refinement and validation statistics

	antibody 1 - 9N5Y - AEL31302/AEL31311	antibody 2 - 9N5Z - AMB38310/AMB38599
**Data collection and processing**		
Microscope	TFS Glacios	TFS Glacios
Voltage (keV)	200	200
Camera	TFS Falcon 4i	TFS Falcon 4i
Collection mode	Counting	Counting
Magnification	190,000x	190,000x
Pixel size at detector (Å)	0.718	0.718
Total electron exposure (e–/Å2)	45	44.84
Number of EER frames	40	40
Defocus range (μ m)	-0.8 to -1.6	-0.8 to -1.6
Automation software	EPU	EPU
Micrographs collected (no.)	4,628	3,976
Micrographs used (no.)	4,457	3,295
Initial particle images (no.)	626,964	641,551
Final particle images (no.)	85,692	142,446
Symmetry	C1	C3
Map resolution (masked/unmasked Å)	3.7/3.8	3.0/3.1
FSC threshold	0.143	0.143
**Refinement**		
Initial model used (PDB code)	7T3D	7T3D
Refinement package	Phenix RSR	Phenix RSR
Model resolution (Å)	3.77	3.16
FSC threshold	0.5	0.5
EMRinger score	3.08	3.32
CC (mask)	0.80	0.77
Model composition		
Non-hydrogen atoms	13,754	13,702
Protein residues	1728	1724
Ligands	10	14
R.m.s. deviations		
Bond lengths (Å)	0.005	0.002
Bond angles (°)	0.996	0.583
Validation		
MolProbity score	1.39	1.61
Clashscore	3.37	12.41
Poor rotamers (%)	0.00	0.00
Ramachandran plot		
Favored (%)	96.14	98.3
Allowed (%)	3.86	1.70
Disallowed (%)	0.00	0.00
Cβ outliers (%)	0.00	0.00
CaBLAM outliers (%)	2.95	2.6

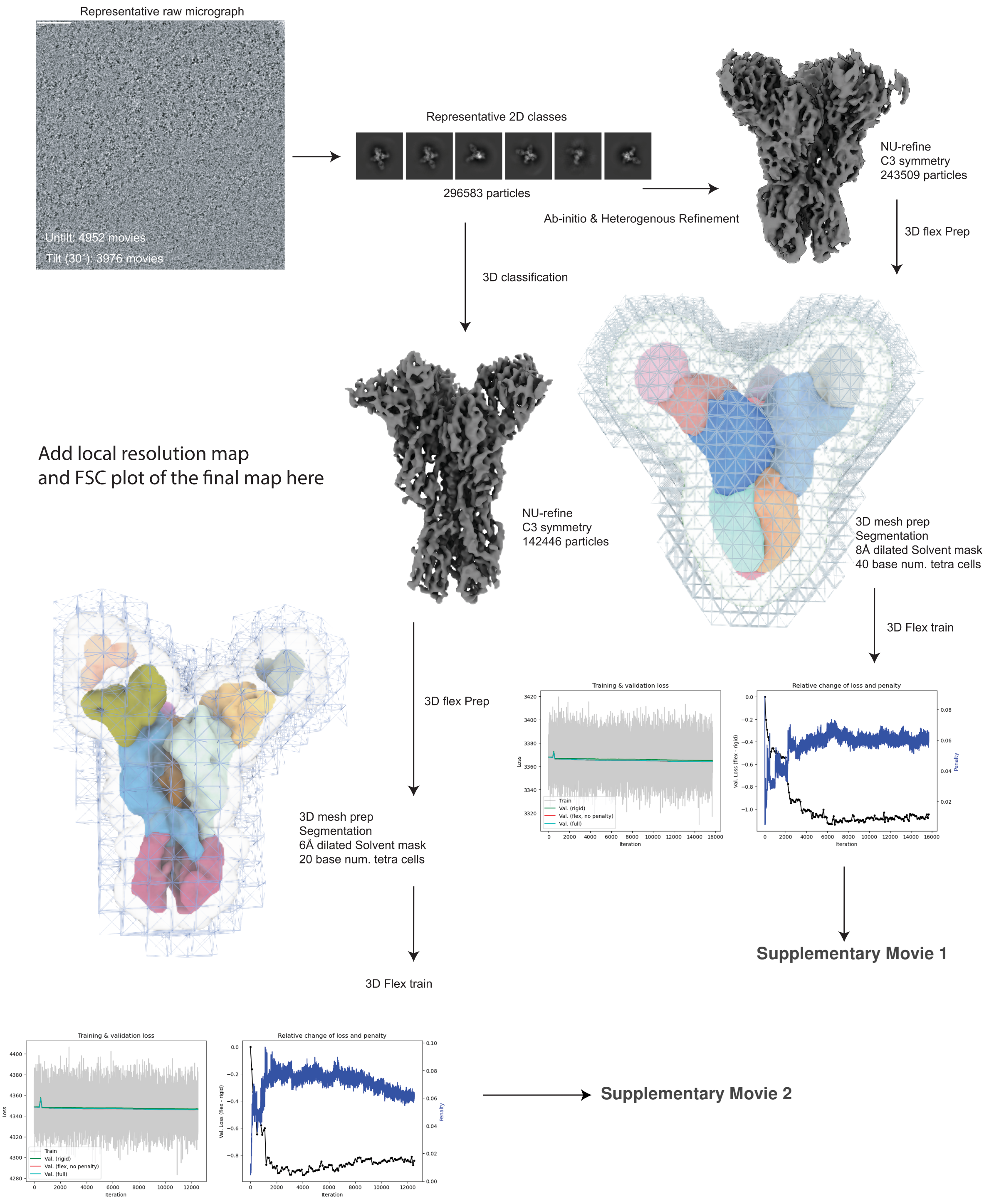

Figure S2: Workflow of the flexibility analysis in cryo-EM experiments. Figure detailing the 3D flex workflow described in the methods section of this manuscript.

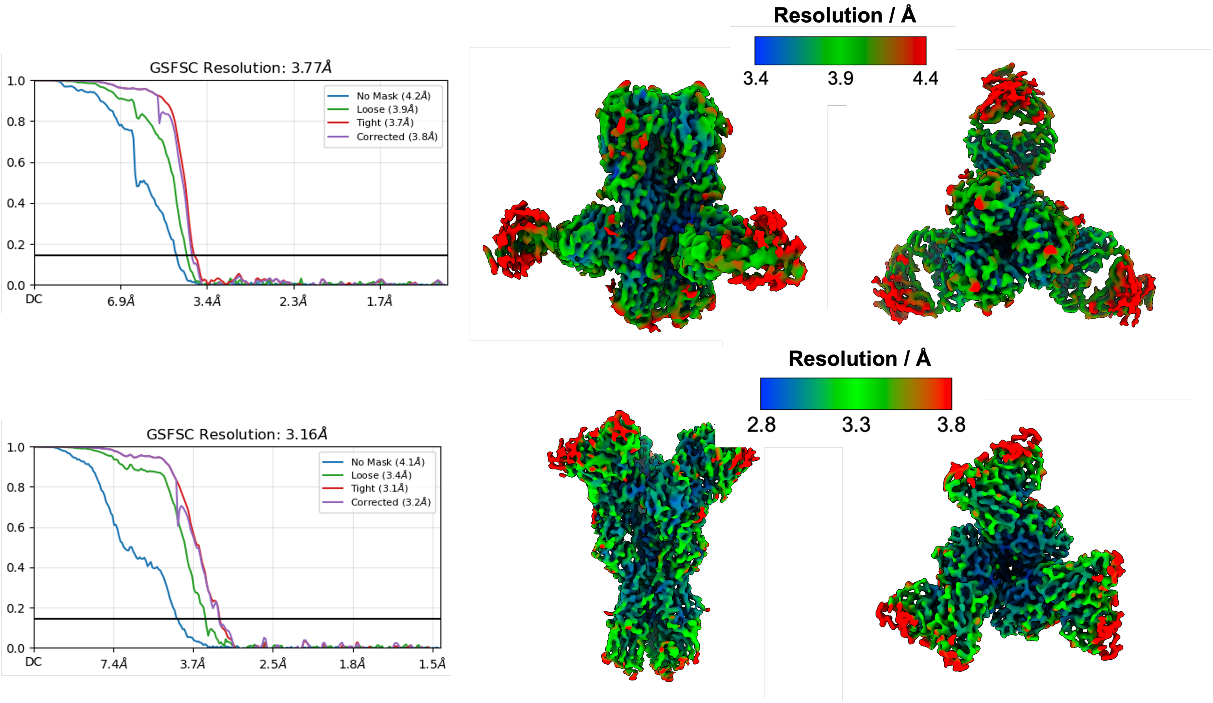

Figure S3: Local resolution maps for antibody 1 / 9N5Y (top) and antibody 2 / 9N5Z (bottom).

S2 Supplementary results

S2.1 CDR3 secondary structure

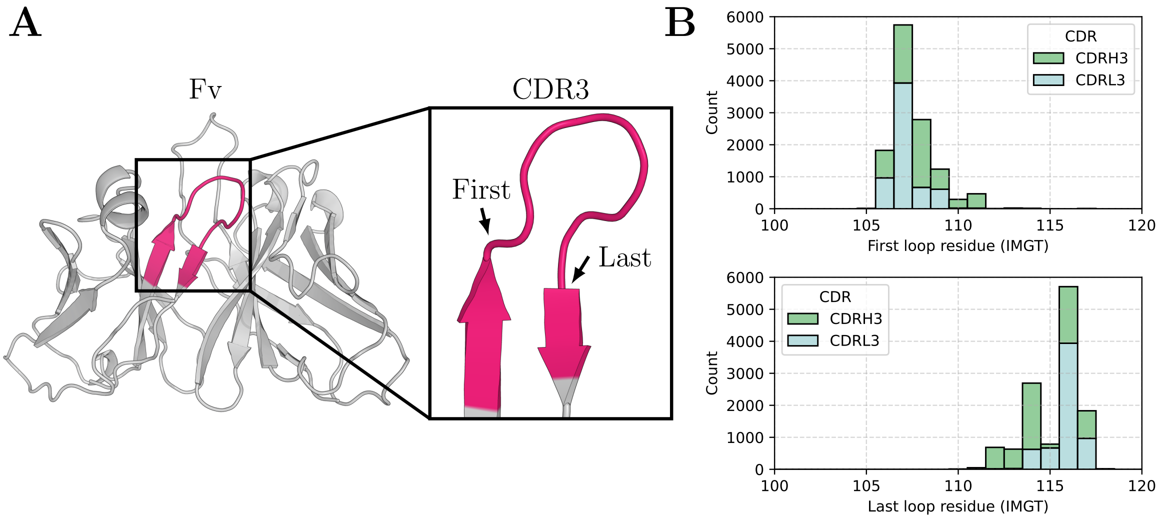

Figure S4: Antibody and TCRs CDR3 are loops bounded by two antiparallel β-strands. A) The variable domains (Fvs) adopt an immunoglobulin fold which consist of 9 antiparallel β-strands. CDR3s, defined by IMGT residues 105 - 117 (highlighted in pink), connect the two C-terminal strands of the Fv. The start (105) and end (117) residues are located on the strands and the majority of the CDR3 forms a loop motif. The first and last residue in the loop motif are highlighted. B) Histogram showing the distribution of antibody variable domain residues (IMGT numbered) that occur as the first and last residue of the loop motif. The majority of loops start at IMGT residue 107 and end at IMGT residue 116. Unless stated otherwise, we define a CDR3 as IMGT residue 107-116 throughout the paper.

S2.2 ALL-conformations

S2.2.1 ALL-conformations statistics

This section provides additional statistics for ALL-conformations. The distributions of loop length and the number of structures available for loops are shown in Figure S5 & S6. Correlation between flexibility and loop length is shown in Table S4. The number of loops labelled as 'flexible' and 'rigid' in each set is shown in Table S5

Table S4: Correlation of CDR length and flexibility

Set	Original dataset (alignment on CDR)	Alternative dataset (alignment on Fv)
CDRH3	0.13	0.11
CDRL3	0.34	0.21
CDRB3	0.36	0.16
CDRA3	0.12	-0.03

R values of the point biserial correlation of the number of residues in the CDR and binary labels rigid (0) and flexible (1).

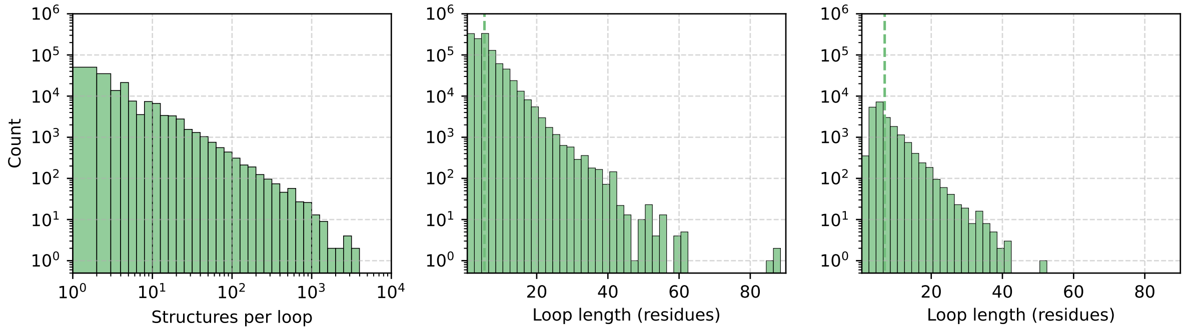

Figure S5: Distributions of ALL-conformations PDB set. The left column shows a histogram of the number of available structures for each loop in ALL-conformations (N=1.2e6). The middle column shows the distribution of loop length for all loops in ALL-conformations (N=1.2e6). The right column shows the distribution of loop length for only those loops with determined flexibility (i.e. rigid and flexible in Table S5, N=20,814). The vertical dashed lines indicate the mean of a distribution.

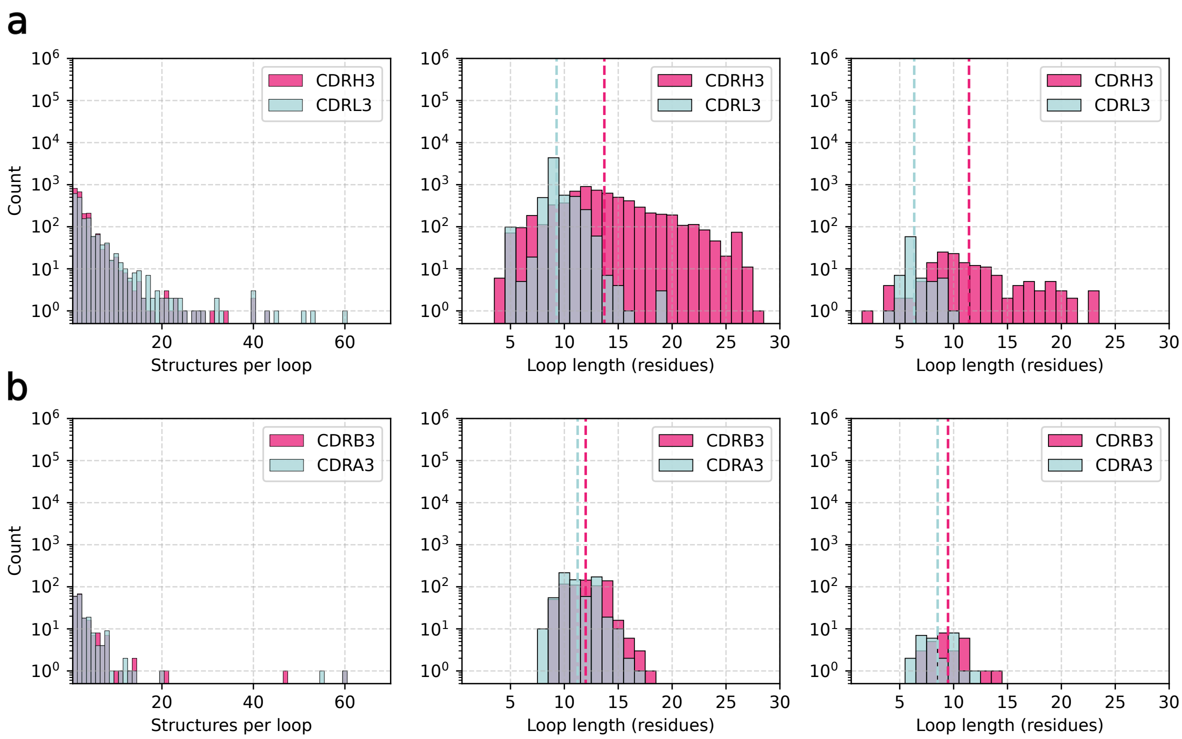

Figure S6: Distributions of ALL-conformations CDR sets. a) Antibody CDRH3 and CDRL3 sets, b) TCR CDRB3 and CDRA3 sets. The left column shows a histogram of the number of available structures for each loop in ALL-conformations. The middle column shows the distribution of loop length for all loops in ALL-conformations. The right column shows the distribution of loop length for only those loops with determined flexibility (i.e. rigid and flexible in Table S5). The vertical dashed lines indicate the mean of a distribution. The number of plotted data points is shown in Tables 1 & S5.

Table S5: Conformational flexibility in ALL-conformations

Dataset	Unknown flexibility	Flexible - multiple conformations	Rigid - single conformation	Total loops with determined flexibility
PDB set	78,000	4289	16,525	20,814
Antibodies				
CDRH3	2325	97	55	152
CDRL3	2282	15	69	84
TCRs				
CDRB3	207	21	6	27
CDRA3	206	21	6	27

S2.2.2 Alternative definition of loop flexibility

In the main sections of this manuscript, we made several choices to determine the conformational flexibility of loops in ALL-conformations. Structures in the PDB set were initially grouped by sequence identity of the loop, whereas structures in the CDR3 sets were grouped by sequence identity of the Fv. Structural similarity was then calculated by the RMSD of loop residues after structural alignment on the loop.

In this section, a different approach to determine the conformational flexibility was explored. In the PDB set structures were grouped by sequence identity of the loop and two additional anchor residues on either of its sides. Structural similarity was then calculated by the RMSD of the loop after structural alignment on the anchor residues.

Antibody and TCR structures were grouped by Fv identity as in the original dataset. To calculate structural similarity, structures were aligned on the framework regions of the chain the CDR is located. We chose to align on the entire framework region rather than anchor residues (as for the PDB set) to reduce noise in the alignment. In certain cases the β-strand located on the C-terminal side of the CDR3 is not resolved well in crystal structures due to its proximity to the end of the chain. When choosing to align on anchor residues only the alignment can therefore show inaccuracies.

A conformation was defined, as described in the methods section, by clustering using an agglomerative clustering with complete linkage and 1.25 Å distance threshold. We recalculated the flexibility of loops in ALL-conformations using this alternative definition of flexibility. Summary statistics for are shown in Table S6.

Table S6: Conformational flexibility in ALL-conformations using alternative definitions

Dataset	Unknown flexibility	Flexible - multiple conformations	Rigid - single conformation	Total loops with determined flexibility
PDB set	128,000	18,526	15,927	34,453
Antibodies				
CDRH3	2241	187	49	236
CDRL3	2239	66	61	127
TCRs				
CDRB3	193	35	6	41
CDRA3	197	31	5	36

S2.3 Extended evaluation of ITsFlexible performance

S2.3.1 Consistency of predictions from multiple structures of the same loop

Throughout this manuscript, ITsFlexible performance is evaluated by making a prediction for each available crystal structure containing the loops in the test set. The mean prediction score across all structures containing the same loop is then used to calculate performance metrics. In this section, we investigate the consistency of the predictions when using different structures of the same loop as input. Figure S7 shows the standard deviation of the ITsFlexible prediction score across all structures containing the same loop. This value is generally small suggesting consistent predictions.

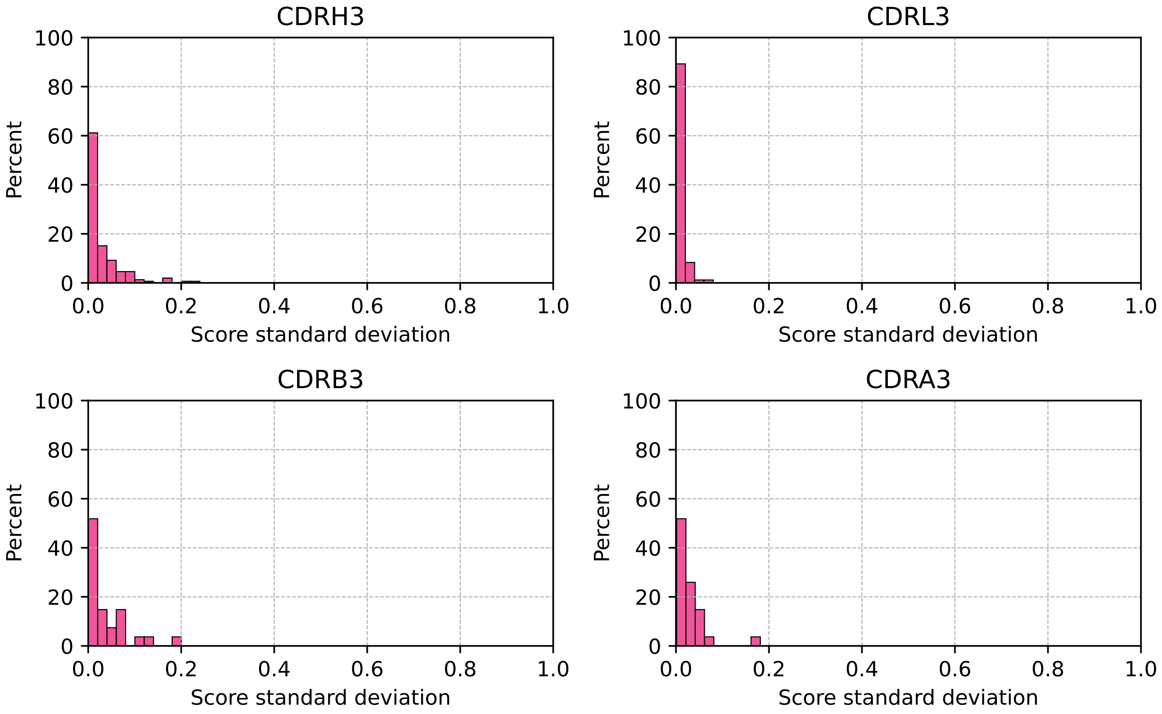

Figure S7: Standard deviation of ITsFlexible prediction scores when making predictions using multiple crystal structures containing the same loop as input. Number of data points plotted for CDRH3: N=147, CDRL3: N=84, CDRB3: N=27, CDRA3: N=27.

S2.3.2 Predictions from structural models

Table S7: Comparison of AF2 and IB model accuracy and impact on ITsFlexible predictions

	CDRH3 (N=147)		CDRL3 (N=84)		CDRB3 (N=27)		CDRA3 (N=27)	
	RMSD	MAE score	RMSD	MAE score	RMSD	MAE score	RMSD	MAE score
Xtal v AF2	3.2 Å	0.073	1.4 Å	0.014	4.9 Å	0.064	5.3 Å	0.120
Xtal v IB	1.8 Å	0.049	0.8 Å	0.011	1.6 Å	0.051	1.8 Å	0.067

The RMSD columns indicate the average C-alpha RMSD of CDR residues after alignment on framework residues between crystal structure and structural models across the CDR test sets. The MAE score columns show the mean absolute error in the ITsFlexible prediction when using a structural model compared to the crystal structure as input.

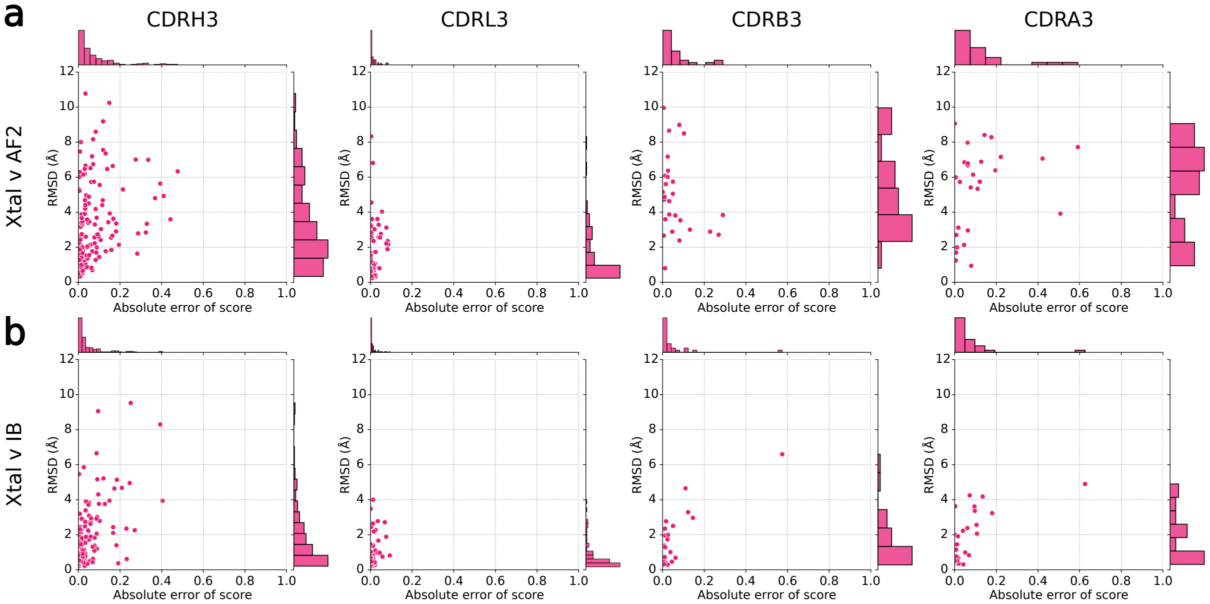

Figure S8: Impact of model accuracy on ITsFlexible prediction. Analysis for (a) AF2 and (b) IB predictions. For the four CDR test sets the RMSD of a structural model is plotted against the absolute error of the ITsFlexible prediction score (i.e. the absolute difference in prediction score when using a structural model instead of the crystal structure as input). Number of data points plotted for CDRH3: N=147, CDRL3: N=84, CDRB3: N=27, CDRA3: N=27.

S2.3.3 Performance with secondary structure-based CDR3 definitions

Throughout the analysis in the main sections of this manuscript we defined CDR3s as IMGT residues 107-116. This range was shown to align with the secondary structure of the loop for the majority of antibodies (Figure S4). Here, we assessed the impact of defining each CDR3 exactly by its secondary structure. We used the DSSP algorithm (Kabsch & Sander, 1983) to identify the exact numbering of the first residue after the immunoglobulin F-strand and the last residue before the G-strand and extracted the region in between as the CDR3. We refer to this definition as the DSSP CDR3.

CDRH3 and CDRL3 test sets were created with conformational flexibility recalculated over DSSP CDR3 definitions and ITsFlexible performance was evaluated (Table S8). Similar performance is achieved for the DSSP and our standard CDR3 definitions. Due to the simplicity of

CDR3s being defined by the same IMGT numbers in all antibodies across the dataset, we chose to use the 107-116 definition throughout the manuscript.

Table S8: DSSP defined CDR test set performance.

METHOD	CDRH3		CDRL3	
	DSSP (N=141)	IMGT 107-116 (N=147)	DSSP (N=84)	IMGT 107-116 (N=84)
RANDOM	0.62	0.65	0.19	0.18
BASELINES				
SOLVENT EXPOSURE	0.66	0.69	0.25	0.32
LENGTH	0.64	0.69	0.29	0.36
COMBINED	0.73	0.72	0.38	0.37
ITSFLEXIBLE				
CRYSTAL STRUCTURE	**0.82**	**0.82**	**0.52**	**0.49**

The performance of the methods on CDR test sets is evaluated by the area under the precision-recall curve (PR AUC). The best performance achieved for each test set is highlighted in bold.

S2.3.4 Performance with alternative definition of flexibility

ITsFlexible was retrained on the dataset with alternative definitions of flexibility as described in Section S2.2.2. This model was parametrised similar to the model described in the methods section. The context distance threshold was increased to 30 Å and the edge threshold distance to 20 Å to provide the model with information of a larger structural context.

Model performance was evaluated on the PDB and the CDR test sets (Figure S9). The data splits were created based on sequence identity. The PDB test set did not contain any loops with more than 80% sequence identity across the loop and anchor residues to length matched loops in the training and validation sets. The CDR test sets did not contain any loops with more than 80% aligned sequence identity to any loop (not restricted to matching loop length) in the training and validation sets. Baselines and alternative workflows were performed as outlined in the methods. We made a small modification of the AF2 MSA subsampling to make the workflow more consistent with the here used definition of flexibility. Flexibility in the structural ensembles was calculated by loop RMSD after alignment on the framework regions of the corresponding chain. ITsFlexible achieves good preformance on all five datasets indicating the models ability to detect flexibility signals independent of choices made when calculating structural flexibility.

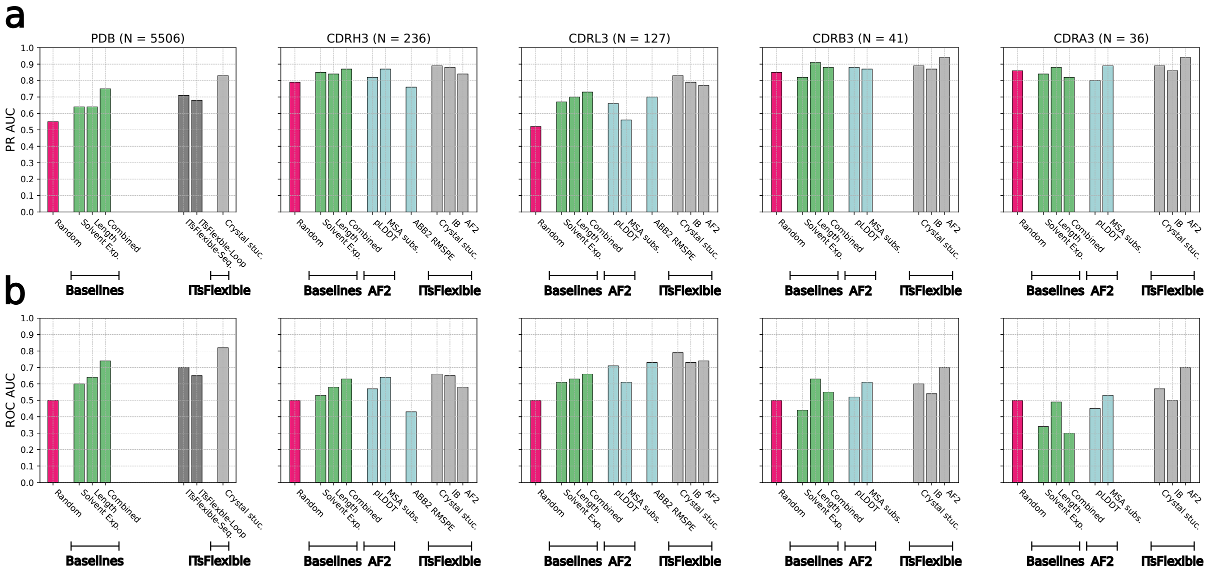

Figure S9: ITsFlexible performance with alternative definitions of flexibility. Classification on the five test sets is evaluated with metrics of (a) PR AUC and (b) ROC AUC. ITsFlexible performance from inputs of crystal structures, ImmuneBuilder (IB) and AF2 models (light grey) is compared to random classification (red), three biophysical baselines (green) and three zero-shot models based on outputs of protein structure prediction tools (blue). Additionally, for the PDB test set we evaluated two ITsFlexible versions with input ablations (dark grey). Exact PR AUC and ROC AUC values are presented in Tables S11 & S12.

S2.3.5 Impact of varying RMSD thresholds on datasets and model performance

In the main sections of this manuscript, we defined a loop conformation as a structural cluster where the pairwise RMSD between any two members is below a threshold of 1.25 Å. This threshold was chosen based on previous work suggesting 1.25 Å as an optimal threshold for functional clustering. Our definition of a conformation should, therefore, most accurately reflect distinct functional states.

In this section, we analysed how different values of the threshold impact the proportion of labels in the datasets. At low thresholds (1 Å) the highest number of flexible and lowest number of rigid loops are observed (Figure S10). Increasing the threshold leads to a large reduction of flexible loops accompanied by a small increase in rigid loops. At a value of 2.5 Å all CDR test sets, except for H3, contain less than five flexible examples.

In a next step, we investigated how varying RMSD threshold and the consequent proportion of labels affects the predictive performance of ITsFlexible. We chose 1 Å and 2 Å as two additional values to evaluate the model. For values below 1 Å the resolution of the experimental structure may make the determined labels less reliable and for values above 2 Å the limited number of flexible loops in the test set would hinder detailed analysis of predictive performance. We relabelled our datasets using these new RMSD thresholds and retrained and evaluated ITsFlexible on these labels. The model architecture and training procedure was kept identical for this experiment.

When using the new RMSD thresholds, ITsFlexible retains high accuracy at predicting loop flexibility (Figure S10). Measured by PR AUC the model outperforms the biophysical baselines and zero-shot classifiers in nearly all CDR test sets. The only exception is the CDRA3 set where

AF2 based zero-shot predictors come top in both cases. As observed for our standard RMSD threshold (1.25 Å), ITsFlexible is the only method that consistently achieves high predictive accuracy across all test sets.

When comparing the classifier performance at the two thresholds, we observed slightly better results at 2 Å. The metrics achieved by ITsFlexible tend to show a larger increase over random classification than at the 1 Å threshold (Figure S10). Looking at the distribution of labels in the datasets, this may perhaps be counterintuitive. A better balance of rigid and flexible loops in the 1 Å datasets would, in theory, be more favourable for model training. However, we speculate that labels are less noisy at higher thresholds which may improve classification.

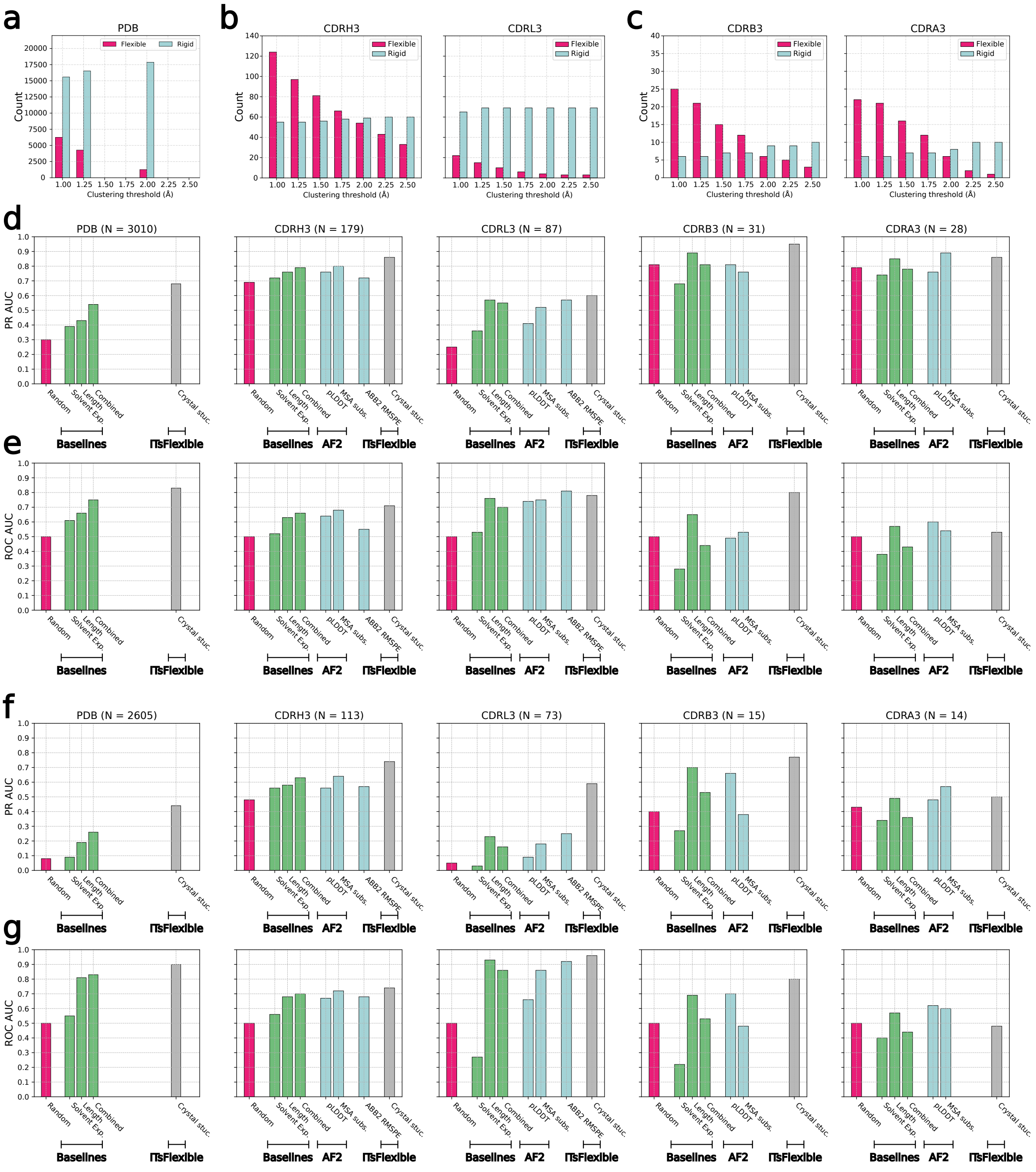

Figure S10: Impact of varying RMSD thresholds on datasets and model performance. a-c) Number of loops labelled as rigid and flexible as a function of the RMSD threshold used to define a conformation. The PDB set is shown in (a), antibody CDRs in (b) and TCR CDRs in (c). d-e) ITsFlexible performance evaluated on the test sets using a 1 Å RMSD threshold in metrics of PR AUC (d) and ROC AUC (e). Exact values of the metrics are presented in Tables S13 & S14. f-g) ITsFlexible performance evaluated on the test sets using a 2 Å RMSD threshold in metrics of PR AUC (f) and ROC AUC (g). Exact values of the metrics are presented in Tables S15 & S16. ITsFlexible performance from inputs of crystal structures (grey) is compared to random classification (red), three biophysical baselines (green) and three zero-shot models based on outputs of protein structure prediction tools (blue).

S2.3.6 Supplementary tables

Table S9: PR AUC values for test set classification

Method	PDB (n = 2845)	CDRH3 (n = 147)	CDRL3 (n = 84)	CDRB3 (n = 27)	CDRA3 (n = 27)
Random	0.23	0.65	0.18	0.78	0.78
Baselines					
Solvent Exposure	0.29	0.69	0.32	0.63	0.73
Length	0.35	0.69	0.36	0.90	0.85
Combined	0.46	0.72	0.37	0.80	0.78
AlphaFold2					
pLDDT	0.45	0.71	0.32	0.81	0.75
MSA subsampling	-	0.76	0.48	0.74	**0.89**
ABB2 rmspe	-	0.70	0.39	-	-
ITsFlexible-sequence	0.47	-	-	-	-
ITsFlexible-loop	0.46	-	-	-	-
ITsFlexible					
Crystal structure	**0.62**	**0.82**	0.49	**0.97**	0.87
ImmuneBuilder	-	0.81	**0.55**	0.93	0.83
AlphaFold2	-	0.75	0.40	0.96	0.85

The best performance achieved for each test set is highlighted in bold.

Table S10: ROC AUC values for test set classification

Method	PDB (n = 2845)	CDRH3 (n = 147)	CDRL3 (n = 84)	CDRB3 (n = 27)	CDRA3 (n = 25)
Random	0.50	0.50	0.50	0.50	0.50
Baselines					
Solvent Exposure	0.60	0.53	0.53	0.23	0.36
Length	0.69	0.60	0.75	0.74	0.58
Combined	0.76	0.63	0.72	0.47	0.43
AlphaFold2					
pLDDT	0.77	0.63	0.75	0.55	0.60
MSA subsampling	-	0.68	0.81	0.57	**0.65**
ABB2 rmspe	-	0.61	0.81	-	-
ITsFlexible-sequence	0.76	-	-	-	-
ITsFlexible-loop	0.77	-	-	-	-
ITsFlexible					
Crystal structure	**0.84**	**0.71**	0.87	**0.89**	0.61
ImmuneBuilder	-	0.69	**0.88**	0.79	0.52
AlphaFold2	-	0.61	0.81	0.86	0.59

The best performance achieved for each test set is highlighted in bold.

Table S11: PR AUC values for test set with alternative definition of flexibility

Method	PDB (n = 5506)	CDRH3 (n = 236)	CDRL3 (n = 127)	CDRB3 (n = 41)	CDRA3 (n = 36)
Random	0.55	0.79	0.52	0.85	0.86
Baselines					
Solvent Exposure	0.64	0.85	0.67	0.82	0.84
Length	0.64	0.84	0.70	0.91	0.88
Combined	0.75	0.87	0.73	0.88	0.82
AlphaFold2					
pLDDT	-	0.82	0.66	0.88	0.80
MSA subsampling	-	0.87	0.56	0.87	0.89
ABB2 rmspe	-	0.76	0.70	-	-
ITsFlexible-sequence	0.71	-	-	-	-
ITsFlexible-loop	0.68	-	-	-	-
ITsFlexible					
Crystal structure	**0.83**	**0.89**	**0.83**	0.89	0.89
ImmuneBuilder	-	0.88	0.79	0.87	0.86
AlphaFold2	-	0.84	0.77	**0.94**	**0.94**

The best performance achieved for each test set is highlighted in bold.

Table S12: ROC AUC values for test set with alternative definition of flexibility

Method	PDB (n = 5506)	CDRH3 (n = 236)	CDRL3 (n = 127)	CDRB3 (n = 41)	CDRA3 (n = 36)
Random	0.50	0.50	0.50	0.50	0.50
Baselines					
Solvent Exposure	0.60	0.58	0.61	0.44	0.34
Length	0.64	0.58	0.63	0.63	0.49
Combined	0.74	0.63	0.66	0.55	0.30
AlphaFold2					
pLDDT	-	0.57	0.71	0.52	0.45
MSA subsampling	-	0.64	0.61	0.61	0.53
ABB2 rmspe	-	0.43	0.73	-	-
ITsFlexible-sequence	0.70	-	-	-	-
ITsFlexible-loop	0.65	-	-	-	-
ITsFlexible					
Crystal structure	**0.82**	**0.66**	**0.79**	0.60	0.57
ImmuneBuilder	-	0.65	0.73	0.54	0.50
AlphaFold2	-	0.58	0.74	**0.70**	**0.70**

The best performance achieved for each test set is highlighted in bold.

Table S13: PR AUC values for test set with 1 Å flexibility threshold

Method	PDB (n = 3010)	CDRH3 (n = 179)	CDRL3 (n = 87)	CDRB3 (n = 31)	CDRA3 (n = 28)
Random	0.30	0.69	0.25	0.81	0.79
Baselines					
Solvent Exposure	0.39	0.72	0.36	0.68	0.74
Length	0.43	0.76	0.57	0.89	0.85
Combined	0.54	0.79	0.55	0.81	0.78
AlphaFold2					
pLDDT	-	0.76	0.41	0.81	0.76
MSA subsampling	-	0.80	0.52	0.76	**0.89**
ABB2 rmspe	-	0.72	0.57	-	-
ITsFlexible					
Crystal structure	**0.68**	**0.86**	**0.60**	**0.95**	0.86

The best performance achieved for each test set is highlighted in bold.

Table S14: ROC AUC values for test set with 1 Å flexibility threshold

Method	PDB (n = 3010)	CDRH3 (n = 179)	CDRL3 (n = 87)	CDRB3 (n = 31)	CDRA3 (n = 28)
Random	0.50	0.50	0.50	0.50	0.50
Baselines					
Solvent Exposure	0.61	0.52	0.53	0.28	0.38
Length	0.66	0.63	0.76	0.65	0.57
Combined	0.75	0.66	0.70	0.44	0.43
AlphaFold2					
pLDDT	-	0.64	0.74	0.49	**0.60**
MSA subsampling	-	0.68	0.75	0.53	0.54
ABB2 rmspe	-	0.55	**0.81**	-	-
ITsFlexible					
Crystal structure	**0.83**	**0.71**	0.78	**0.80**	0.53

The best performance achieved for each test set is highlighted in bold.

Table S15: PR AUC values for test set with 2 Å flexibility threshold

Method	PDB (n = 2605)	CDRH3 (n = 113)	CDRL3 (n = 73)	CDRB3 (n = 15)	CDRA3 (n = 14)
Random	0.08	0.48	0.05	0.40	0.43
Baselines					
Solvent Exposure	0.09	0.56	0.03	0.27	0.34
Length	0.19	0.58	0.23	0.70	0.49
Combined	0.26	0.63	0.16	0.53	0.36
AlphaFold2					
pLDDT	-	0.56	0.09	0.66	0.48
MSA subsampling	-	0.64	0.18	0.38	**0.57**
ABB2 rmspe	-	0.57	0.25	-	-
ITsFlexible					
Crystal structure	**0.44**	**0.74**	**0.59**	**0.77**	0.50

The best performance achieved for each test set is highlighted in bold.

Table S16: ROC AUC values for test set with 2 Å flexibility threshold

Method	PDB (n = 2605)	CDRH3 (n = 113)	CDRL3 (n = 73)	CDRB3 (n = 15)	CDRA3 (n = 14)
Random	0.50	0.50	0.50	0.50	0.50
Baselines					
Solvent Exposure	0.55	0.56	0.27	0.22	0.40
Length	0.81	0.68	0.93	0.69	0.57
Combined	0.83	0.70	0.86	0.53	0.44
AlphaFold2					
pLDDT	-	0.67	0.66	0.70	**0.62**
MSA subsampling	-	0.72	0.86	0.48	0.60
ABB2 rmspe	-	0.68	0.92	-	-
ITsFlexible					
Crystal structure	**0.90**	**0.74**	**0.96**	**0.80**	0.48

The best performance achieved for each test set is highlighted in bold.

Table S17: ITsFlexible performance on the MD test set

Method	CDRH3 (n = 19)		CDRL3 (n = 19)	
	ROC AUC	PR AUC	ROC AUC	PR AUC
Random	0.50	0.84	0.50	0.37
ITsFlexible				
max	0.96	0.99	0.77	0.74
mean	0.85	0.97	0.58	0.50

Two different approaches were used to classify the flexibility of MD simulated antibodies with ITsFlexible. In both cases we made a prediction for each representative structure extracted from MD. For our standard evaluation protocol we use the maximum score to calculate metrics. We also report metrics when using the mean prediction score. The first approach achieved better metrics for both test sets. Taking the mean had little effect on CDRH3s and larger effects on CDRL3s which seems to be due to the narrower distribution of scores.

S2.4 ABodyBuilder2

S2.4.1 ABB2 retraining

The original ABB2 (Abanades et al., 2023) was trained on a version of SAbDab (Schneider et al., 2022) downloaded in July 2021. As SAbDab was also used to create ALL-conformations and consequently the antibody CDR flexibility test sets, there is a substantial overlap with the ABB2 training set (Figure S18). To avoid data leakage, ABB2 was retrained using the original training protocol on a dataset with the ALL-conformations overlap removed. Antibodies with 100% sequence identity in CDRH3 or CDRL3 regions to a data point in the CDRH3 or CDRL3 flexibility test sets were removed from the training set. This reduced the number of training set antibodies from 5669 to 4469. The validation and test set were not changed. The accuracy of the retrained ABB2 is compared to the original ABB2 on a benchmark of 34 non-redundant antibody structures from the SAbDab (Table S19). The retrained ABB2 retained high accuracy at predicting antibody structures.

Table S18: Overlap of antibody CDR test sets and ABB2 training set

	CDRH3	CDRL3
Total in test set	147	84
ABB2 training overlap*	127	76

*Number of CDR3 sequences that also appear in the ABB2 training set.

Table S19: Comparison of ABB2 and retrained ABB2 on benchmark

Method	CDRH1	CDRH2	CDRH3	FwH	CDRL1	CDRL2	CDRL3	FwL
ABB2 original	0.86	0.74	2.61	0.74	0.52	0.38	0.87	0.56
ABB2 retrained	0.87	0.75	2.93	0.76	0.54	0.35	0.92	0.58

The mean RMSD (in Å) of the prediction to the crystal structure across the benchmark set is shown for the six CDRs and the framework regions according to IMGT definitions.

S2.4.2 Flexibility classification with ABB2

In this section, we show results of using the original ABB2 (trained by Abanades et al. (2023)) for flexibility classification and highlight that due to data leakage and dataset biases the performance is not representative. Flexibility classification based on RMSPEs obtained from structures predicted with the original ABB2 performs substantially better than using the retrained ABB2 (Table S20). This boost in performance is linked to biases in the CDR flexibility test sets. When labelling CDRs as rigid or flexible, we introduced a requirement that a rigid CDR needs to appear in at least five separate PDB structures (see methods). Therefore, CDRs labelled as rigid tend to be represented by more copies in the ABB2 training set as the ones labelled to be flexible (Figure S11).

We further observed a negative correlation between the number of times a CDR occurs in the ABB2 training set and its RMSPE (Figure S11). This finding can be explained by the way that ABB2 calculates the PE score. ABB2 predicts four structures for each antibody, the PE score is then calculated as the diversity between these structures (Abanades et al., 2023). The more often an antibody appears in the training set, the more it is weighted in the training loss. This

makes it more likely that the antibody structure is predicted with high accuracy and reduces the diversity between the four structures. The correlation disappears for the retrained ABB2 which shows that the correlation is indeed an artifact of the training set.

To highlight the importance of this bias, we predicted flexibility simply based on the number of times an antibody appears in the original ABB2 training set (Table S20). This predictor, based on data bias only, performs closer to the original ABB2 than to the random baseline.

Table S20: Flexibility classification with ABB2

Method	CDRH3 (n = 147)	CDRL3 (n = 84)
Random	0.65	0.18
ITsFlexible	0.82	0.49
ABB2 rmspe		
Retained	0.70	0.39
Original	0.81	0.62
N structures in ABB2 training set*	0.74	0.41

*Logistic regression model with input corresponding to the number of times a given CDR occurs in the ABB2 training.
The performance of the methods on CDR test sets is evaluated by the area under the precision-recall curve (PR AUC).

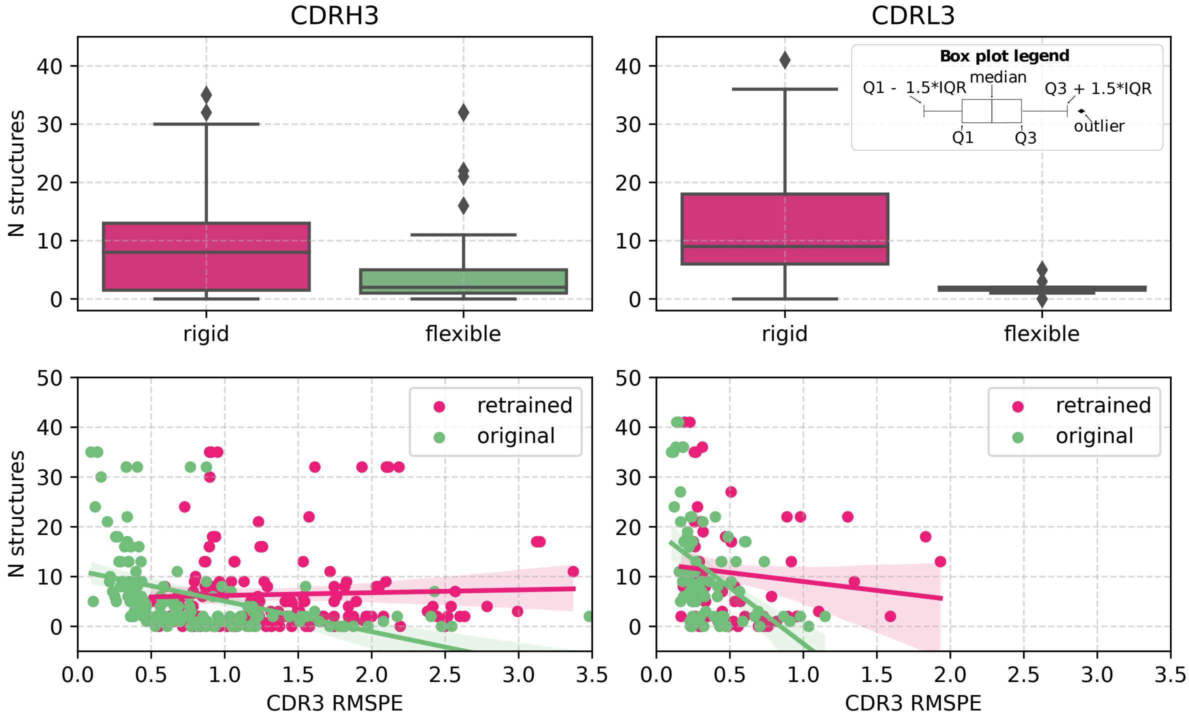

Figure S11: Biases in the antibody CDR3 flexibility classification test sets. top) Boxplot of the number of structures appearing in the original ABB2 training set for CDRs labelled as flexible and rigid. Rigid CDRs tend to be represented more often in the training set than flexible structures. The box shows the quartiles of the distribution, the whiskers extend to 1.5 times the inter-quartile range (IQRs) of the lower and upper quartile and outliers are shown as points. Number of data points plotted for CDRH3-rigid: N=59, CDRH3-flexible: N=54, CDR3-rigid: N=69, CDRL3-flexible: N=4. bottom) The number of structures in the original ABB2 training set is plotted against the CDR3 RMSPE for the retrained and the original ABB2. A linear regression is fit to the data points. For the original ABB2 there is a moderate negative correlation (CDRH3: Pearson $R = -0.42$, CDRL3: Pearson $R = -0.47$) between the number of training set structures and the RMSPE of a CDR. For the retrained ABB2 there is no correlation (CDRH3: Pearson $R = 0.06$, CDRL3: Pearson $R = -0.12$). Data points plotted for CDRH3: N=113, CDRL3: N=73.

S2.5 Case study antibodies for cryo-EM

Table S21: ITsFlexible and zero-shot classifier predictions of cryo-EM case study antibodies

ID	ITsFlexible prediction	ITsFlexible score		AF2 pLDDT		AF2 MSA RMSD		ABB2 RMSPE	
		Value	Pctile	Value	Pctile	Value	Pctile	Value	Pctile
1 - 9N5Y	rigid	0.02	29	71	73	3.13	100	0.90	33
2 - 9N5Z	flexible	0.76	100	68	81	2.80	100	2.20	89
3	flexible	0.60	97	71	67	0.51	6	1.42	54

Pctile indicates the percentile of the value compared to CDRs with the same label across the ALL-conformations CDRH3 test set. The percentile is calculated so that a higher number indicates a more flexible prediction. For the ITsFlexible score, AF2 MSA RMSD and ABB2 RMSPE higher values are associated with increased flexibility and the 100th percentile is set to the highest value observed in the test set. For the AF2 pLDDT a lower value is associated with increased flexiblity and the 100th percentile is set to the lowest value observed in the test set.

References

Abanades, B., Wong, W. K., Boyles, F., Georges, G., Bujotzek, A., and Deane, C. M. ImmuneBuilder: Deep-Learning models for predicting the structures of immune proteins. *Communications Biology*, 6:575, May 2023. ISSN 2399-3642. doi: 10.1038/s42003-023-04927-7. URL https://www.nature.com/articles/s42003-023-04927-7.

Berman, H. M. The Protein Data Bank. *Nucleic Acids Research*, 28(1):235–242, January 2000. ISSN 13624962. doi: 10.1093/nar/28.1.235. URL https://academic.oup.com/nar/article-lookup/doi/10.1093/nar/28.1.235.

Kabsch, W. and Sander, C. Dictionary of protein secondary structure: pattern recognition of hydrogen-bonded and geometrical features. *Biopolymers*, 22(12):2577–2637, December 1983. ISSN 0006-3525. doi: 10.1002/bip.360221211. URL https://onlinelibrary.wiley.com/doi/10.1002/bip.360221211.

Kingma, D. P. and Ba, J. Adam: A Method for Stochastic Optimization, 2014. URL https://arxiv.org/abs/1412.6980.

Satorras, V. G., Hoogeboom, E., and Welling, M. E(n) Equivariant Graph Neural Networks, February 2022. URL http://arxiv.org/abs/2102.09844. arXiv:2102.09844 [cs, stat].

Schneider, C., Raybould, M. I. J., and Deane, C. M. SAbDab in the age of biotherapeutics: updates including SAbDab-nano, the nanobody structure tracker. *Nucleic Acids Research*, 50 (D1):D1368–D1372, January 2022. ISSN 0305-1048, 1362-4962. doi: 10.1093/nar/gkab1050. URL https://academic.oup.com/nar/article/50/D1/D1368/6431822.
